# Results from UKALL60+, a phase 2 study in older patients with untreated acute lymphoblastic leukemia

**DOI:** 10.1002/hem3.88

**Published:** 2024-06-06

**Authors:** Bela Patel, Amy A. Kirkwood, Clare J. Rowntree, Krisztina Z. Alapi, Emilio Barretta, Laura Clifton‐Hadley, Tom Creasey, SooWah Lee, David I. Marks, Anthony V. Moorman, Nicholas Morley, Pip Patrick, Zaynab Rana, Anita Rijneveld, John A. Snowden, Adele K. Fielding

**Affiliations:** ^1^ Centre for Haemato‐Oncology, Barts Cancer Institute Queen Mary University of London London UK; ^2^ CR UK and UCL Cancer Trial Centre, UCL Cancer Institute, UCL London UK; ^3^ Cardiff and Vale UHB Cardiff Wales UK; ^4^ Hematology, UCL Cancer Institute London UK; ^5^ Leukemia Research Cytogenetics Group, Translational and Clinical Research Institute Newcastle University Newcastle Upon Tyne UK; ^6^ United Bristol Healthcare Trust Bristol UK; ^7^ Royal Hallamshire Hospital Sheffield Teaching Hospital NHS Foundation Trust Sheffield UK; ^8^ Erasmus Medical Centre Rotterdam Netherlands

Poor outcome for older patients with ALL has multiple attributions, including a higher incidence of high‐risk genetic features,^1^ and comorbidities as well as treatment intolerance.^2^, ^3^ The phase 2 clinical trial UKALL60+ (NCT01616238) was a collaboration between the UK National Cancer Research Institute Adult ALL Group and the Haemato‐Oncology Foundation for Adults in the Netherlands (HOVON) to study treatment choices, quality of life (QoL) and outcomes in older patients with ALL. UKALL60+ offered four treatment “pathways”: pathway A for *BCR::ABL1*+ ALL and pathways B, C, and D offering three choices of intensity for *BCR::ABL1* negative ALL (Intensive, Intensive‐plus and Non‐Intensive, respectively), to be selected by investigator and patients. A registration‐only choice (Pathway E) was also available. Details of treatment regimens are given in Figure S1. There were no exclusions for any comorbidities. The primary endpoint was complete remission (CR) after a 2‐phase induction. Secondary endpoints included event‐free survival (EFS) and overall survival (OS), the predictive value of MRD (Ig/TCR quantification, EuroMRD criteria),^4^ patient‐reported outcomes, and the relationship between the baseline characteristics (Charlson index. ECOG, Karnofsky and Chemotherapy Risk Assessment Scale for High‐Age Patients [CRASH] scores) and treatment option chosen.

Between January 2013 and November 2018, 121 eligible patients, median age 69 (interquartile range [IQR]: 65–73, range: 55–83), of whom 107 had B‐ALL and 14 T‐ALL, were recruited at 34 sites (Table S1). Baseline characteristics are shown in Table 1 alongside the characteristics of the 65 patients aged over 60 years that were recruited to the contemporaneous UKALL14 trial, age 25–65 years. A consort diagram is shown in Figure S2.

**Table 1:** Baseline characteristics and main survival outcomes by pathway.

	Pathway A *BCR::ABL1*+	Pathway B Intensive	Pathway C Intensive‐plus	Pathway D Non‐intensive	Pathway E Registration only	All	UKALL 14 cohort
	*N* = 25	*N* = 51	*N* = 9	*N* = 21	*N* = 15	*N* = 121	*N* = 65
Baseline characteristic	*N* (%)	*N* (%)	*N* (%)	*N* (%)	*N* (%)	*N* (%)	*N* (%)
Demographics							
Age (years), median (IQR) Range	66 (63−69) 55−83	67 (65−72) 56−76	67 (62−70) 58−70	73 (70−78) 64−82	71 (66−78) 61−83	69 (65−73) 55−83	62 (61−64) 60–65
		B vs. C *p* = 0.41, B vs. D *p* = 0.001, C vs. D *p* = 0.094					
Age group, *N* (%)							
55–64	7 (28)	11 (21.6)	3 (33.3)	2 (9.5)	3 (20)	26 (21.5)	57 (87.7)
65–74	13 (52)	36 (70.6)	6 (66.7)	11 (52.4)	8 (53.3)	74 (61.2)	8 (12.3)
75–84	5 (20)	4 (7.8)	0	8 (38.1)	4 (26.7)	21 (17.4)	0
Sex, *N* (**%**)							
Female	14 (56)	28 (54.9)	2 (22.2)	10 (47.6)	9 (60.0)	63 (52.1)	34 (52.3)
Male	11 (44)	23 (45.1)	7 (77.8)	11 (52.4)	6 (40.0)	58 (47.9)	31 (47.7)
ECOG, *N* (**%**)							
0–2	23 (92)	51 (100)	94 (100)	21 (100)	14 (92.9)	108 (97.3)	63 (100)
3–4	2 (8)	0	0	0	1 (7.1)	3 (2.7)	0
Missing	0	0	0	0	0	0	1
		B vs C *p* = 0.98, B vs. D *p* = 0.081, C vs. D *p* = 0.26					
Disease characteristics							
Cell type, *N* (**%**)							
Precursor‐B‐cell disease	23 (95.8)	40 (78.4)	7 (87.5)	20 (100)	12 (92.3)	102 (87.9)	59 (90.8)
T‐cell disease	1 (4.2)	11 (21.6)	1 (12.5)	0	1 (7.7)	14 (12.1)	6 (9.2)
Missing	1	0	1	1	2	5	
WBC risk group,^a^ *N* (%)							
Standard risk	17 (70.8)	45 (90)	7 (87.5)	19 (90.5)	11 (73.3)	99 (83.9)	47 (72.3)
High risk	7 (29.2)	5 (10)	1 (12.5)	2 (9.5)	4 (26.7)	19 (16.1)	18 (27.7)
Missing	1	1	1	0	0	3	0
Genetic subgroup at diagnosis,^b^ *N* (%)							
High risk	0	1 (2)	1 (12.5)	2 (10)	1 (6.7)	5 (4.3)	15 (25.4)
Standard risk	0	25 (51)	3 (37.5)	13 (65)	6 (40)	47 (40.2)	4 (6.8)
*BCR::ABL1*+	24 (96)	0	0	0	4 (26.7)	28 (23.9)	21 (35.6)
Very high risk	0	12 (24.5)	3 (37.5)	5 (25)	3 (20)	23 (19.7)	5 (8.5)
Test failed or missing	0	2	1	1	0	4	6
IADL Score, median (IQR)	28 (27−28)	28 (27−28)	27.5 (26−28)	27 (23−28)	27.5 (21−28)	28 (27−28)	‐
		B vs. C *p* = 0.34, B vs. D *p* = 0.041, C vs. D *p* = 0.94					
$Crash Score ‐ Combined Score, median (IQR)	7 (5−8)	7 (5−8)	6 (5−8)	8 (7−8)	7.5 (5−9)	7 (5−8)	‐
Intermediate high/high risk (7+)	13 (52)	31 (60.8)	33 (33.3)	18 (85.7)	8 (57.1)	73 (60.8)	‐
		B vs. C *p* = 0.16, B v.s D *p* = 0.053, C vs. D *p* = 0.008					
Charleson Index, median (IQR)	6 (5−7)	6 (5−6)	5 (5−5)	6.5 (6−7)	7 (5−9)	6 (5−7)	‐
High risk (7+)	8 (32)	8 (16.7)	0	9 (45)	8 (53.3)	33 (27.5)	
		B vs. C *p* = 0.34, B vs. D *p* = 0.014, C vs. D *p* = 0.027					
Outcomes							
Induction CR % (70% CI)	92 (82.1–97.2)	70.6 (62.6–77.6)	55.6 (33.6–75.9)	47.6 (34.5–61)	66.7 (47.6–82.2)	69.5 (64.5–74.1)	‐
MRD neg CR rate^c ^% (incl. unknowns)	5 (20)	13 (25.5)	2 (22.2)	1 (4.8)	2 (13.3)	23 (19)	22 (34.9)
Event‐free survival	19 events	45 events	5 events	18 events	13 events	100 events	55 events
*N* relapses	15	26	2	8	6	56	
Median % (95% CI) mo.	13.7 (7.6−31.5)	12.9 (9.1–18.5)	12.4 (0.6–NR)	9.1 (4.6–11.4)	13.4 (4.7−21.4)	12.2 (9.8–15)	9.6 (6.1–13.3)
1‐yr EFS % (95% CI)	56 (34.8–72.7)	54.9 (40.3–67.3)	55.6 (20.4–80.5)	25.2 (9.2–45.1)	66 (36.5–84.3)	51.1 (41.8–60)	43.3 (31–55)
3‐yr EFS% (95% CI)	27.0 (11.5–45.3)	16.5 (7.7–28.3)	41.7 (10.9–70.8)	15.1 (3.8–33.7)	7.4 (0.5–28.1)	18.8 (12.2–26.5)	20.5 (11.5–31.3)
Overall survival	18 deaths	43 deaths	4 deaths	18 deaths	13 deaths	96 deaths	51 deaths
Median (95% CI) mo.	23 (11.8–54.3)	18.1 (11.3–24.2)	NR	10.9 (5–13.2)	16.7 (4.7–24.8)	15.8 (12.7–19.5)	13.1 (8.6–19.2)
1‐yr OS% (95% CI)	71.4 (49.2−85.2)	64.7 (50–76.1)	53.3 (17.7–79.6)	45.2 (23.3–64.9)	66 (36.5–84.3)	62 (52.6–70.1)	52.8 (39.9–64.2)
3‐yr OS% (95% CI)	33.0 (15.5–51.8)	20.8 (10.8–33.0)	55.3 (17.7–79.6)	15.1 (3.8–33.6)	14.7 (2.4–37.3)	23.2 (15.9–31.4)	25.2 (15.2–36.5)

Abbreviation: ECOG, Eastern Cooperative Oncology Group.

^$^ Modified from the literature which suggests a score of 5, but 2 points for ALL, therefore this has been inflated to 7.

^a^ High risk: ≥30 for B‐cell and ≥100 for T‐cell.

^b^ High risk: KMT2A fusions; (1) very high risk: low hypodiploidy or near triploidy, complex [more than five abnormalities) or JAK‐STAT abnormalities and standard risk: all other patients.

^c^ Includes patients who were MRD negative after phase 1.

Fifty‐one of 81 (63%) patients with *BCR::ABL1* negative disease were allocated to pathway B, 11% (9/81) to pathway C, and the remaining 26% (21/81) to pathway D. At a median follow‐up: 65.9 months (IQR: 38.1–80.9), CR rate after two phases of induction, was achieved by 92% (70% confidence interval [CI]: 82.1–97.2) on pathway A, 70.6% (70% CI: 62.6–77.6) on pathway B, 55.6% (70% CI: 33.6–75.9) on pathway C and 47.6% (70% CI: 34.5%–61%) of those on pathway D. No participant achieved CR on study later than end of induction. Molecular remission occurred in 5/25 (20%; A), 13/51 (25.5%; B), 2/9 (22.2%; C), and 1/21 (4.8%; D) with data available. Only 26/121 (21.5%) patients achieved molecular remission at any point. The relationship between MRD and outcome at the three study timepoints is given in Table S2.

Ninety‐six deaths were reported; 32 patients died without achieving CR (22/32, primary cause, ALL). Fifty‐six patients died after relapse and eight died in CR (four from infection, three from second malignancies [small cell lung cancer, AML, and CMML] and one unknown). Survival data are shown in Table 1, with the corresponding Kaplan–Meier survival curves in Figure 1. At a median follow‐up of 65.9 months (IQR: 38.1–80.9), the estimated 1‐year EFS rates were: pathway A: 56.0% (95% CI: 34.8–72.7), pathway B: 54.9% (95% CI: 40.3–67.3), pathway C: 55.6% (95% CI: 20.4–80.5) and pathway D: 25.2% (95% CI: 9.2–45.1). The corresponding OS was: pathway A: 71.4% (95% CI: 49.2−85.2), pathway B: 64.7% (95% CI: 50.0–76.1), pathway C: 53.3% (95% CI: 17.7–79.6), and pathway D: 15.1% (95% CI: 3.8–33.6). The higher initial CR rate for patients with *BCR::ABL positive* ALL (pathway A) did not result in a markedly better 1‐year EFS or OS than the *BCR::ABL1* negative participants, regardless of pathway. The 3‐year EFS (95% CI) were: pathway A: 27.0% (11.5–45.3), pathway B: 16.5% (7.7–28.3), pathway C: 41.7% (10.9–70.8), and pathway D: 15.1% (3.8–33.70) and OS (95% CI): pathway A: 33.0% (15.5–51.8), pathway B: 20.8% (10.8–33.0), pathway C: 55.3% (17.7–79.6) and pathway D: 15.1% (3.8–33.6). Three‐year EFS for those achieving CR was 27.6% (18.2–37.8) and for those achieving molecular remission was 33.6% (16.4–51.7), as shown in Figure S3. Only 14 patients with T‐ALL were recruited, with no discernable difference in outcome to B‐ALL (Table S3). A description of the pathway E, registration‐only cohort is given in the supplement.

**Figure 1:**
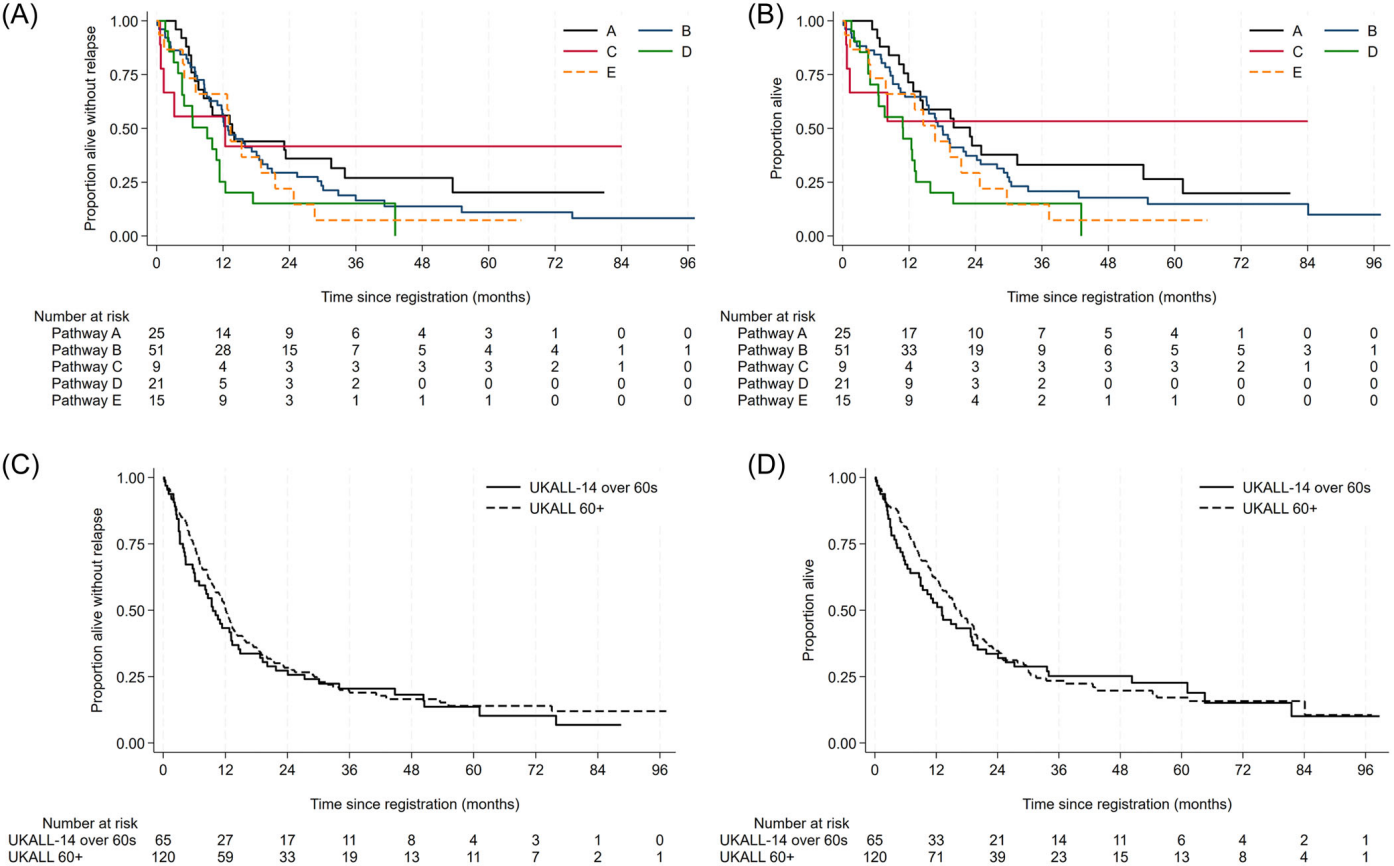
Kaplan–Meier survival curves. (A, B) Kaplan–Meier survival curves by UKALL60+ treatment pathways with (A) event‐free survival (EFS) and (B) overall survival (OS). (C, D) Kaplan–Meier survival curves for overall outcomes among UKALL60+ cohort and the UKALL14 60‐ to 65‐year‐old participants with (C) EFS and (D) OS. Note that one pathway E UKALL60+ registrant received the UKALL14 protocol and is included in the UKALL14 cohort only.

Adverse events (AEs), duration of hospitalization, treatment cessation by phase of therapy, are shown in Tables S4 and S5. Five of 121 (4.1%) patients (none of whom had achieved CR) suffered a fatal AE (three pathway C and one each pathways B and D), the causes being one cardiac arrest, three lung infections, and one febrile neutropenia. Grade 3/4 events were common (98/102; 96%), particularly infections (88/102; 86.3%). Patients in pathway C experienced significantly more grade 3/4 AEs during induction 1 (medians 16.0 [IQR: 8.0–20.5]) and induction 2 (15.0 [9.0–17.5]) compared to patients in pathways A (9.0 [6.0–11.0], *p* = 0.045 [induction 1] and 6.0 [3.0–8.0], *p* = 0.030 [induction 2]) and D (6.5 [5.5–10.0], *p* = 0.026 [induction 1] and 5.5 [3.5–8.5], *p* = 0.026 [induction 2]). More events were also seen for pathway B (10.0 [8.0–13.0], *p* = 0.023) than pathway D. Only 21/106 (19.8%) patients completed all protocol treatment. Discontinuation was highest during inductions 1 and 2; 27/106 (25.5%) and 11/106 (10.4%) mainly due to refractory/relapsed disease (19/38; 50%). Relapsed/refractory ALL was also the main reason for discontinuation of therapy at other timepoints (33/47; 70.2%) across all arms. Only five of 106 (4.7%) overtly stopped therapy due to toxicity.

A comparison of patient characteristics across the pathways is shown in Table 1. Participants on pathway D were significantly older than those on pathway B (median 73 years [IQR: 70–78] vs. 67 [IQR: 62–70], *p* = 0.0001), and had greater comorbidity; 9/21 (45%) with a Charleston Index of 7 or more in pathway D compared to only 8/51 (16.7%) in pathway B. The greater frailty of the pathway D group was also evident when comparing baseline QoL measures and comorbidities, with significantly lower physical functioning compared to pathway B; medians 60.0 (IQR: 53.3–80) versus 86.7 (IQR: 66.7–100), *p* = 0.014 (Tables S6 and S7). No patient with a Charleston index score of 7 or above was allocated to pathway C (*p* = 0.013). Major, age‐associated comorbidities were common across the entire study cohort and included cardiac disease 27/121 (22.3%), diabetes 17/121 (14.0%), hypertension 39/121 (32.2%), and other cancer 22/121 (18.1%), eight of which were previous breast cancer and seven previous hematological malignancy.

Significant differences were seen in duration of inpatient stay, analysed by percentage of total treatment period spent in hospital (*p* = 0.026). Patients receiving pathways B and C spent more treatment‐time in hospital compared to pathways A and D, with the effect most pronounced during induction (*p* = 0.0001) where pathway B and C participants were inpatients for 62.1% (46.3–96.7) and 75.8% (68.8–83.0), respectively compared to pathway A and D participants at 22.8% (IQR: 9.8–55.6) and 31.1% (IQR: 14.5–51.5), respectively. QoL was compared by pathway—summarized in supplementary results. We saw no indication that the least intensive pathway D provided a better QoL, with scores for some scales numerically lower than those of pathways A–C (Figure S4A–D). Any decreases in QoL from baseline were generally seen at the end of induction phases, with improvements in the FACT scores seen by the end of consolidation 1 and maintenance 1 (Table S8). Physical function scores, as assessed by QLQ‐C30, remained reduced throughout while sensory and motor neuropathy scores increased at later points during therapy.

We compared the EFS and OS of UKALL60+ cohort with that of the 65 patients aged 60–65 years, treated on the full intensity adult ALL trial UKALL14 in an overlapping recruitment timeframe. Unsurprisingly, patients in UKALL14 had lower ECOG scores (40.0% vs. 60.9% ECOG 0, *p* = 0.0068). Fewer had baseline comorbidities (67.7% vs. 85.0%, *p* = 0.0059)—particularly notable for cardiac morbidities (6.2% vs. 22.5% *p* = 0.0046). Although CR rates were higher; UKALL14 55/63 (87.3%) vs. UKALL60+ 82/118 (69.2%), as shown in Table 1, EFS and OS rates at 3 years were 20.5% (11.5–31.3) and 25.2% (15.2–36.5) for UKALL14 and 18.8% (12.2–26.5) and 23.2% (15.9–31.4) for UKALL60+ (Figure 1C,D). However, the type of events differed, with only 18/55 (32.7%) being relapse, 9/55 deaths without remission, and 26/55 (47.2%) deaths in remission in UKALL14 compared to 56/95 (58.9%), 32/95 (33.7%), and 7/95 (7.4%) respectively, for UKALL60+. Among the UKALL14 60–65 year‐old cohort, 28/65 had received allo‐SCT, resulting in death in remission in 3/28 (46%).^5^ Among patients treated on pathway B, there was no difference in outcome between patients with high/very high‐risk versus standard‐risk genetics (EFS HR: 1.35 [0.68–2.67], *p* = 0.39) whereas there was a difference in outcome by genetic risk for patients aged 60–65 years old treated on UKALL14 (HR: 2.59 [1.12–5.98], *p* = 0.026).

Our survival data are broadly commensurate with data from a GMALL cohort of similar median age^6^ where OS at 3 years was 32%. However, the UKALL60+ population were in worse health overall; 27.5% had a Charlson Index >7 compared to the GMALL cohort, with only 11% scoring >3, commensurate with the GMALL exclusion criteria for comorbidities. By contrast to the GMALL early death rate of 14%, we observed a low treatment‐related mortality. UKALL60+ treatments were successfully planned to minimize harm, but this did not improve OS.

It was unexpected that the 3‐year EFS and OS of 20.5% and 25.2% for those aged 60–65 years treated on UKALL14 data did not differ from that of the UKALL60+ recruits, with completely overlapping survival curves (Figure 1). The outcomes are similar to those reported in an EBMT study of 418 patients aged over 55 years receiving alloSCT; 5‐year LFS of 34% but a 51% nonrelapse mortality.^7^ Taken together, these data suggest even the most intensive treatments including alloSCT do not generate excellent outcomes for most older patients with ALL. We were surprised to see no evidence of better QoL for the recipients of the least intensive pathway D, despite a significant reduction in length of hospital stay.

In summary, the UKALL60+ pathways proved safe for initial cytoreduction, but the survival outcomes in this representative population of older patients with ALL was unsatisfactory. Nonchemotherapy approaches should be employed at the earliest opportunity for this patient group. QoL should always be measured, as the investigators assumptions do not necessarily reflect patients' experience.

## AUTHOR CONTRIBUTIONS

**Bela Patel**: Trial design, trial management group member, manuscript writing. **Amy A. Kirkwood**: Trial design, lead statistician, data analysis, manuscript writing. **Clare J. Rowntree**: Trial management group member, site principal investigator. **Krisztina Z. Alapi**: Central lab manager, specimen processing, MRD analysis. **Emilio Barretta**: Central analysis of genetics. **Laura Clifton‐Hadley**: Trial management group member, lead trial co‐ordination. **Tom Creasey**: Central analysis of genetics. **SooWah Lee**: Specimen processing, MRD analysis. **David I. Marks**: Trial management group member, site principal investigator. **Anthony V. Moorman**: Trial management group member, central coordination of genetics. **Nicholas Morley**: Trial management group member, site principal investigator. **Pip Patrick**: Trial management group member, senior trial coordination. **Zaynab Rana**: Trial management group member, trial coordination. **Anita Rijneveld**: Site principal investigator, lead for HOVON. **John A. Snowden**: Trial management group member, quality of life lead. **Adele K. Fielding**: Trial concept and design, trial management group chair, trial chief investigator, manuscript writing, trial funding.

## CONFLICT OF INTEREST STATEMENT

The authors declare no conflict of interest.

## FUNDING

Cancer Research UK CRUK/A13920 to AKF, CRUK/A21019 to AKF/AVM, and an unrestricted educational support grant from Jazz Pharma.

## Supporting information

Supporting information.

## Data Availability

The data that support the findings of this study are available from the corresponding author upon reasonable request.
