# Supplementary material for: Results from UKALL60+, a phase 2 study in older patients with untreated acute lymphoblastic leukemia
Source: Hemasphere. 2024 Jun 6;8(6):e88. doi: 10.1002/hem3.88 (PMC11154829; doi:10.1002/hem3.88)
Supplement: Supplementary file 1 — Supporting information. [file HEM3-8-e88-s001.docx]

**Results From UKALL60+, A Phase 2 Study In Older Patients With Untreated Acute Lymphoblastic Leukaemia: supplementary methods and results**

**Methods**

*Study Eligibility and Design*

Eligible patients were aged over 60 years (or over 55 years but unfit for UKALL14 or HOVON100 trials) with newly diagnosed ALL. There were no exclusion criteria for poor organ function, prior malignancy or performance status. Ethical approval was obtained from the UK national research ethics committee (London-Bloomsbury). All patients gave written, informed consent, according to the Declaration of Helsinki. The four study treatments included one regimen for *BCR::ABL1*+ patients (Pathway A), and a choice of Intensive (Pathway B), vs Intensive- plus (Pathway C, HOVON117-based) vs Non-intensive (Pathway D). Regimen was chosen per physician/patient preference with reason for the choice documented. A “registration only” pathway (E) allowed any suitable treatment: outcome data and specimens were collected. The Charlson index, the Instrumental Activities of Daily Living scale (IADL), CRASH, ECOG and Karnofsky performance status were collected at diagnosis. QoL was also assessed at baseline, after each treatment phase and 3-monthly during maintenance using the EORTC QLQ–C 30, EORTC CIPN, FACT-Leu, and FACIT-FATIGUE scales. Adverse events (AE), recorded after each treatment phase until maintenance, were graded according to Common Terminology Criteria for Adverse Events (CTCAE) v4.0 and follow-up was required yearly after treatment completion. The trial opened as an observational non-IMP study, but on 27/01/2016 after 72 patients had been recruited to pathways A-D, the UK competent authority re-reviewed the protocol and required an amendment to re-open as an IMP study on 4^th^ August 2016. HOVON could not subsequently reactivate the trial.

The sponsor was University College London, coordination was done by the CR UK and UCL Cancer Trials Centre.  The trial was funded by Cancer Research UK (CR UK/A13920) with additional funding from an educational grant from Jazz Pharmaceuticals. Funders had no role in the study design, data collection, data analysis, data interpretation or writing of the report. This study was registered with ClinicalTrials.gov, NCT01616238 and an Independent Data Monitoring Committee reviewed its safety and efficacy data yearly.

*UKALL 60+ Treatment*

A summary of all UKALL 60+ chemotherapy treatments is provided in Supplementary figure 1.  Full details are given in the protocol, available on request. A 5-7 day (d) steroid pre-phase of dexamethasone 6mg/m^2^/d capped at 10mg/d was permitted prior to trial registration. Formal assessment of response was documented after phase 1 and 2 induction after intensification (for patients on the pathway B arm only); and after consolidation 1 (pathways A, C and D) by conventional light microscopy. Patients who did not achieve CR following induction could continue with protocol therapy if they were gaining clinical benefit. Prophylaxis against fungal infections, PJP and HSV/VZV re-activations and was as per local policy. Growth factor support was recommended although not mandated. Minimal residual disease (MRD) testing was performed after each of the first three treatment cycles, in a central, EuroMRD-accredited laboratory using standardised techniques of Q-PCR quantification of patient-specific immunoglobulin heavy chain/T cell receptor rearrangements (*BCR::ABL1* negative ALL) or *BCR::ABL1* fusion gene quantification by RQ-PCR.  MRD was defined as negative, positive, positive outside the quantitative range (POQR) or indeterminate (assay did not reach the required minimum assay sensitivity <10^-4^). Complete MRD response was defined as CR with a negative or POQR MRD result. MRD results were not supplied to centres and did not influence treatment decisions.

*Statistical analysis*

The primary endpoint was investigator-assessed CR rate after two phases of induction treatment. The secondary endpoints were: EFS at 1 year, OS at 1 year, treatment related mortality, CR rate after 1 phase of induction, predictive significance of molecularly determined MRD at three time-points during therapy, tolerability of treatment as determined by occurrence of key adverse effects, duration of in-patient hospitalisation, relationship between performance status/comorbidity and treatment option chosen and QoL measures. The sample size was calculated using an A’Hern single arm design, aiming for a 75% CR rate with 80% power to exclude a rate of 60% or below from the 70% confidence interval. This required 37 patients per pathway for a total of 148 patients in pathways A-D. After opening, pathway selection was very unevenly distributed, so recruitment to a limit of 148 regardless of pathway choice was decided. The trial closed after 106 eligible patients had been recruited to pathways A-D, due to the delays described. It was not our intention to directly compare pathways for efficacy outcomes. Where tests were performed, Chi squared or Fisher’s exact tests were used for discrete data and Wilcoxon Mann Whitney or Kruskal Wallis tests for continuous data. EFS, (events: relapse and death) and OS were analysed using Kaplan-Meier survival analysis, with time measured from the date of registration until the date of the first event. Patients without an event were censored at the date last seen. Repeated measures mixed models were used to assess QoL. Change from baseline was assessed at 4 timepoints, with pathways A-D also compared at these 4 points. All models were adjusted for the baseline value of each score and p<0.01 was considered statistically significant. A separate cohort of patients aged over 60 years and treated on the contemporaneous UKALL14 trial (for fit adults up to 65 years) were included as a comparator group for efficacy outcomes, although direct statistical comparisons have not been made. Analyses are intention-to-treat unless otherwise specified. Misdiagnoses were excluded from any analysis.  All analyses were performed using STATA v16.1 (STATACorp, Texas).

*Data availability:* data are available from UCL Cancer Trial Centre and can be shared under appropriate ethical conditions after approval by the trial management group.

**Supplementary results:**

**Description of pathway E registrants**

Fifteen patients were recruited to the registration only pathway. Six of 15 were recruited to this pathway entirely due to administrative reasons; 4 were registered after starting treatment: 2 needed urgent intervention, 2 presented before their site was open and registered retrospectively and 2 had a received a steroid pre-phase of longer duration than recommended, making them ineligible for pathways A-D). All 6 patients received treatments similar to these pathways. A further two patients did not want to take part in the “trial” aspects including additional samples, but were treated with a near-identical Pathway A regimen. Another two did not specify reasons beyond “clinician choice” and were treated with a limited induction/maintenance schedule and an identical Pathway B schedule. Four patients chose pathway E because they desired a treatment less intensive than pathway D including one who elected supportive care only and one patient who declined all IV drugs. Finally, one patient opted for treatment on the full intensity UKALL14 trial. It did not appear that the trial enrollment process and trial requirements on study were the main drivers of non-study treatment choice.

The baseline characteristics and outcomes for those treated on pathway E are presented in main Tables 1 and 2. There were no obvious differences in baseline characteristics of pathway E entrants compared to the four study arms.  No conclusions can be drawn about outcomes.

**Comparison of quality of life by pathway**

QOL forms were completed by 91/106 (85.8%) at baseline, but rates of completion dropped sharply as patients discontinued study treatment. Differences from baseline and between treatment pathways were compared at time points collected in all 4 arms: end of induction 1 (60/106;56.6% forms), induction 2 (46/106;42.4%), consolidation 1 (42/106;39.6%) and maintenance cycle 1 (330/106;28.3%). D

**QLQ-C30 questionnaire**

There was no significant change in global health score from baseline to any of the 4 timepoints, nor did we see a significant differences by pathway, with no suggestions that the less intensive pathway D had better QOL (Supplementary figure 2A).

Overall physical functioning was significantly reduced compared to baseline for induction 1 (-15.51 99% CI: (-26.10 to -4.92), p<0.001), induction 2 (-16.05 (99% CI: -27.42 to -4.69), p<0.001) and consolidation 1 (-13.23 (99% CI: -25.21 to -1.24), p=0.004), and had a similar decrease (-13.33 (99% CI: -26.71 to 0.05), borderline significant, p=0.01) after maintenance 1. At almost all timepoints the biggest decreases were seen for pathway D (Supplementary figure 2B), though they did not reach significance. The only significant differences by pathway were seen between pathway A and B at the end of induction 1 (B higher, + 16.53 (99% CI: 0.22 to 32.85), p=0.009) and between B and D at the end of cons 1 (B higher, +31.22 96.39 to 56.06), p=0.001).

The other functional scales showed no significant difference from baseline at the 4 timepoints.

The only two symptom scales which showed differences from baseline were dyspnea which appeared improved in induction 1 (-15.08 (99% CI: -28.38 to -1.78), p=0.003) and consolidation 1 (-18.18 (99% CI: -33.28 to -3.08), p=0.002) and nausea and vomiting, which showed significant increases at the end of induction 1 (16.77 (99% CI: 2.97 to 30.56), p=0.002) and induction 2 (22.48 (99% CI: 7.63 to 37.34), p<0.001) and raised (non-significantly) at consolidation 1 and maintenance. The highest scores were seen for pathway A, which as significantly worse than B (+31.76 (99% CI: 13.15 to 50.36), p<0.001) and D (+34.43 (99%CI: 11.45 to 57.40), p<0.001) at induction 1 and remained significantly higher than B in consolidation 1 (+25.69 (99%CI: 4.26 to 47.12), p=0.002), likely driven by the imatinib given in this pathway. Pathway A also showed other increased gastrointestinal side effects; increased appetite loss compared to D in induction (+33.59 (99%CI: 2.31 to 64.86), p=0.006) and a borderline increase compared to B for diarrhea (+17.21 (99%CI: 0.04 to 34.38), p=0.01) after consolidation 1.

**CIPN-20 (chemotherapy induced peripheral neuropathy)**

Chemotherapy induced peripheral neuropathy was assessed using the CIPN-20 questionnaire. Increases in sensory and motor issues were seen (supplementary figure 3 and supplementary table 2), differences from baseline were higher for sensory effects, and significant at all 4 timepoints (all p<0.01), with motor issues significantly increased at the end of induction 2, consolidation 1 and maintenance cycle 1. There were no significant differences from baseline for autonomic changes. Sensory changes followed a pattern one might have expected with pathway D showing the smallest changes followed by A, B and the C with the largest, though significant differences were only seen between A and B (B higher, +11.5 (99% CI: 0.17 to 22.75), p=0.009) at end of induction 2 and between C and D at end of consolidation 1 (C higher, +27.40 (0.28 to 54.53), p=0.009). There were no significant differences seen by pathway for motor or autonomic changes.

**FACT-G, FACT-LEU and FACIT-Fatigue**

Scales for overall measures broadly showed the same effects with improvements in the FACT-G and both Leukemia and fatigue measures at the two later time points (end of consolidation 1 and maintenance, supplementary table 4). Only emotional wellbeing showed improvements at all timepoints from baseline (all p<0.001). The only differences seen by pathway were better physical well-being for B compared to A at induction 1 (+3.90 (99% CI 0.33 to 7.47), p=0.005) and a better leukemia subscale score for B than D (+13.13 (99% CI: -25.31 to -0.96), p=0.005) at the end of maintenance 1.

**Table S1: Recruitment by site**

| Site | No Patients | Site PI |
| --- | --- | --- |
| University College London /Royal Free Hospitals | 14 | Prof Adele Fielding |
| Royal Hallamshire Hospital, Sheffield | 12 | Dr Nick Morley |
| Bristol Haematology & Oncology Centre, Bristol | 8 | Prof David Marks |
| St James's University Hospital, Leeds | 8 | Dr Richard Kelly |
| University Hospital of Wales, Cardiff | 8 | Dr Clare Rowntree |
| Castle Hill Hospital, Hull | 6 | Dr Simone Green |
| Leicester Royal Infirmary | 6 | Dr Ann Hunter |
| Victoria Hospital Blackpool, Blackpool | 6 | Dr Paul Cahalin |
| St Bartholomew's Hospital, London | 5 | Dr Matthew Smith |
| Churchill Hospital, Oxford | 4 | Dr Andy Peniket |
| Derriford Hospital, Plymouth | 4 | Dr Hannah Hunter |
| James Cook University Hospital, Middlesbrough | 4 | Dr Diane Plews |
| Monklands Hospital, Airdrie | 4 | Dr Lindsay Mitchell |
| St George's Hospital London, London | 4 | Dr Matthias Klammer |
| Christie Hospital, Manchester | 3 | Dr Samar Kulkarni |
| Northwick Park Hospital, Harrow | 3 | Dr Nicki Panoskaltsis/ Dr Vaitsa Katsomitrou |
| Western General Hospital, Edinburgh | 3 | Dr Huw Roddie |
| Arrowe Park Hospital, Wirral | 2 | Dr Barbara Hammer |
| Kings College Hospital, London | 2 | Dr Deborah Yallop |
| Musgrove Park Hospital, Taunton | 2 | Dr Simon Bolam |
| Ninewells Hospital, Dundee | 2 | Dr Sudhir Tauro |
| Poole Hospital, Poole | 2 | Dr Fergus Jack |
| Royal Liverpool Hospital, Liverpool | 2 | Dr Rahuman Salim |
| Whiston Hospital, Prescot | 2 | Dr Toby Nicholson |
| Royal Marsden Hospital | 1 | Dr David Taussig |
| Bradford Royal Infirmary | 1 | Dr Adrian Williams/ Dr Lisa Newton |
| Erasmus MC | 1 | Dr. A.W. Rijneveld |
| Great Western Hospital, Swindon | 1 | Dr Norbert Blesing |
| Raigmore Hospital, Inverness | 1 | Dr Catherine Ogilvie |
| Royal Bournemouth General Hospital, Bournemouth | 1 | Dr Joseph Chacko |
| Russells Hall Hospital, Dudley | 1 | Dr Craig Taylor |
| Salisbury District Hospital | 1 | Dr Jonathan Cullis |
| Sandwell Hospital, West Bromwich | 1 | Dr Yasmin Hasan |
| Torbay District General Hosxpital, Torquay | 1 | Dr Deborah Turner |

**Table S2: Predictive value of MRD at the 3 timepoints**

|  | **Timepoint 1**  End induction 1 | **Timepoint 2**  End induction 2 | **Timepoint 3**  After the first post-induction course |
| --- | --- | --- | --- |
| **N pathways A-D with MRD data** | 49 | 44 | 12 |
| **N neg/POQR (EFS events)** | 8 (7) | 19 (13) | 5 (3) |
| **N pos (EFS events)** | 41 (32) | 25 (19) | 7 (5) |
| **Hazard ratio MRD pos vs negative** | 0.99 (0.44 – 2.26) | 1.55 (0.76 – 3.16) | 2.43 (0.46 – 12.80) |
| **P value** | 0.99 | 0.23 | 0.29 |

**Table S3 -EFS and OS for B and T ALL**

| Pathway | 1-year EFS | 2-years EFS | *3-year EFS | 1-years OS | 2-years OS | 3-years OS |
| --- | --- | --- | --- | --- | --- | --- |
| B-cell | 51.0% (40.9 – 60.2) | 30.4% (21.8 – 339.4) | 21.0% (13.6 – 29.5) | 60.8% (50.6 – 69.5) | 36.3% (27.1 – 45.5) | 25.8% (17.6 – 34.7) |
| **T-cell | 50.0% (22.9 – 72.2) | 14.3% (2.3 – 36.6) | - | 64.3% (34.3 – 83.3) | 21.4% (5.2 – 444.8) | - |

*No patient with T-ALL have been followed -up for 3 years so the 3 year rates are not available

** Only N=14 T-cell (1 pathway A, 11 B, 1 C and 1 reg only) , all but one have had an event - 9 relapses (all later died) and 4 deaths without relapse.

**Table S4: Duration of hospitalisation, treatment cessation by phase of therapy**

|  |  | | | | **Pathway A** | **Pathway B** | **Pathway C** | **Pathway D** |
| --- | --- | --- | --- | --- | --- | --- | --- | --- |
| **Induction 1** | | | | | **N=25** | **N=51** | **N=9** | **N=21** |
|  | Days in hospital, median (IQR)  range | | | | 15 (4 – 23)  0-51 | 28 (17-38)  0-95 | 28.5 (19 – 39)  16-46 | 18 (8-28)  0-48 |
|  | % induction phase 1 in hospital, median (IQR) | | | | 22.9 (9.8 – 55.6) | 62.1 (46.3 – 96.7) | 75.8 (68.8 – 83) | 32.1 (14.5 – 51.5) |
|  | **Stopped during/after this phase, N (%)** | | | | **2 (8)** | **15 (29.4)** | **5 (55.6)** | **5 (23.8)** |
|  |  | | Death | | 0 | 1 | 3 | 0 |
|  |  | | Not fit enough | | 2 | 0 | 0 | 2 |
|  |  | | Patient decision | | 0 | 2 | 0 | 1 |
|  |  | | Refractory disease/failure to achieve remission | | 0 | 8 | 1 | 2 |
|  |  | | Relapse | | 0 | 1 | 0 | 0 |
|  |  | | Switched from B to D | | 0 | 1 | 0 | 0 |
|  |  | | Toxicity | | 0 | 2 | 1 | 0 |
| **Induction 2** | | | | | **N=23** | **N=36** | **N=4** | **N=16** |
|  | Days in hospital, median (IQR)  range | | | | 0 (0-5)  0-51 | 3 (0-12)  0-31 | 1,15,15 and 26 | 0 (0-2)  0-6 |
|  | **Stopped during/after this phase, N (%)** | | | | **3 (12.0)** | **4 (7.8)** | **0** | **4 (19.1)** |
|  |  | | Death | | 0 | 0 | 0 | 1 |
|  |  | | Not fit enough | | 0 | 1 | 0 | 0 |
|  |  | | Refractory disease/failure to achieve remission | | 2 | 2 | 0 | 3 |
|  |  | | For more intensive treatment/SCT | | 1 | 1 | 0 | 0 |
| **Intensification** | | | | | **-** | **N=32** | **-** | **-** |
|  | Days in hospital, median (IQR)  range | | | |  | 10 (7.5-13.5)  3 - 20 |  |  |
|  | **Stopped during/after this phase, N (%)** | | | |  | **2 (3.9)** |  |  |
|  |  | Relapse | | |  | 1 |  |  |
|  |  | To have an SCT | | |  | 1 |  |  |
| **Consolidation 1** | | | | | **N=20** | **N=30** | **N=4** | **N=12** |
|  | Days in hospital, median (IQR)  range | | | | 0 (0-2)  0-6 | 0 (0-1)  0-8 | 0, 0, 6 and 8 | 0 (0—0)  0-11 |
|  | **Stopped during/after this phase, N (%)** | | | | **3 (12.0)** | **2 (3.9)** | **0** | **3 (14.3)** |
|  |  | Relapse | | | 2 | 1 | 0 | 1 |
|  |  | Refractory disease/failure to achieve remission | | | 0 | 1 | 0 | 2 |
|  |  | Toxicity | | | 1 | 0 | 0 | 0 |
| **Consolidation 2** | | | | |  | **N=28** | **N=4** |  |
|  | Days in hospital, median (IQR)  range | | | |  | 0 (0-0)  0-2 | 0,0,4 and 8 |  |
|  | **Stopped during/after this phase, N (%)** | | | |  | **0** | **1 (11.1)** |  |
|  |  | | | Relapse |  | 0 | 1 |  |
| **Consolidation 3** | | | | | **-** | **N=28** | **-** | **-** |
|  | Days in hospital, median (IQR)  range | | | |  | 0 (0-0)  0-2 |  |  |
|  | **Stopped during/after this phase, N (%)** | | | |  | **2 (3.9)** |  |  |
|  |  | | | Relapse |  | 1 |  |  |
|  |  | | | Patient decision |  | 1 |  |  |
| **Maintenance** | | | | | **N=17** | **N=26** | **N=3** | **N=9** |
|  | Months of maintenance, median (IQR)  Range | | | | 9 (6-24)  1-24 | 15 (6-24)  1 – 24 | 24, 26 and 27 | 6 (5.5-24)  1 -24 |
|  | Total days in hospital, median (IQR)  Range | | | | 1 (0-14)  0-48 | 0 (0-3)  0-26 | 2, 6 and 22 | All 0 |
|  | Average days in hospital per month, median (IQR)  Range | | | | 0.04 (0-0.58)  0-8 | 0 (0-0.13)  0-1.6 | 0.08, 0.22, 0.85 | All 0 |
|  | Patients with any time in hospital, N(%) | | | | 8 (53.5) | 8 (32) | 3 (100) | 0 |
|  | **Stopped during this phase, N (%)** | | | | **12** | **16** | **0** | **6** |
|  |  | | | Death | 1 | 1 | 0 | 0 |
|  |  | | | Relapse | 6 | 14 | 0 | 4 |
|  |  | | | Refractory disease/failure to achieve remission | 0 | 0 | 0 | 1 |
|  |  | | | Increasing BCR-ABL | 1 | 0 | 0 | 0 |
|  |  | | | Patient decision | 0 | 0 | 0 | 1 |
|  |  | | | Toxicity | 0 | 1 | 0 | 0 |
|  |  | | | To have an SCT | 4 | 0 | 0 | 0 |
| **Overall treatment** | | | | |  |  |  |  |
| **Completed all trial treatment** | | | | | **5 (20)** | **10 (19.6)** | **3 (33.3)** | **3 (14.3)** |
| **Proportion of all treatment (%), spent in hospital** | | | | | **6 (2.8 – 19.1)** | **14.4 (6.4 – 39.8)** | **24.4 (8.1 – 70.4)** | **8.3 (3.8 – 30.6)** |
|  | | | | |  |  |  |  |

**Table S5 Adverse events**

| **SOC\Adverse event** | | | **Pathway A** | | | **Pathway B** | | | **Pathway C** | | | **Pathway D** | | |
| --- | --- | --- | --- | --- | --- | --- | --- | --- | --- | --- | --- | --- | --- | --- |
|  |  |  | **N=25** | | | **N=49** | | | **N=8** | | | **N=20** | | |
|  |  |  | **N (%)** | | | **N (%)** | | | **N (%)** | | | **N (%)** | | |
|  |  |  | **G1-2** | **G3-4** | **G5** | **G1-2** | **G3-4** | **G5** | **G1-2** | **G3-4** | **G5** | **G1-2** | **G3-4** | **G5** |
|  |  | |  |  |  |  |  |  |  |  |  |  |  |  |
| **Blood and lymphatic system disorders** | | | **6 (24)** | **16 (64)** | **0** | **-** | **37 (75.5)** | **0** | **-** | **7 (87.5)** | **0** | **-** | **13 (65)** | **1 (5)** |
|  | Febrile neutropenia | | - | 3 (12) | 0 | - | 23 (46.9) | 0 | - | 6 (75) | 0 | - | 3 (15) | 1 (5) |
|  | Anaemia | | 6 (24) | 16 (64) | 0 | - | 28 (57.1) | 0 | - | 5 (62.5) | 0 | - | 13 (65) | 0 |
|  | Leucocytosis | | - | 0 | 0 | - | 0 | 0 | - | 0 | 0 | - | 1 (5) | 0 |
| **Cardiac disorders** | | | **-** | **1 (4.0)** | **0** | **-** | **0** | **1 (2.0)** | **3 (37.5)** | **0** | **0** | **-** | **1 (5)** | **0** |
|  | Chest pain - cardiac | | - | 0 | 0 | - | 0 | 0 | - | 0 | 0 | - | 1 (5) | 0 |
|  | Cardiac arrest | | - | 0 | 0 | - | 0 | 1 (2.0) | - | 0 | 0 | - | 0 | 0 |
|  | Myocardial infarction | | - | 1 (4.0) | 0 | - | 0 | 0 | - | 0 | 0 | - | 0 | 0 |
|  | Sinus tachycardia | | - | 0 | 0 | - | 0 | 0 | 2 (25.0) | 0 | 0 | - | 0 | 0 |
| **Gastrointestinal disorders** | | | **16 (64)** | **4 (16.0)** | **0** | **20 (40.8)** | **12 (24.5)** | **0** | **2 (25.0)** | **5 (62.5)** | **0** | **6 (30)** | **3 (15)** | **0** |
|  | Rectal haemorrhage | | - | 0 | 0 | - | 0 | 0 | - | 0 | 0 | - | 1 (5) | 0 |
|  | Dysphagia | | - | 0 | 0 | - | 0 | 0 | - | 0 | 0 | - | 1 (5) | 0 |
|  | Nausea | | 15 (60) | 1 (4) | 0 | 16 (32.7) | 1 (2) | 0 | 4 (50) | 0 | 0 | - | 0 | 0 |
|  | Vomiting | | 15 (60) | 1 (4) | 0 | 13 (26.5) | 1 (2) | 0 | 3 (37.5) | 0 | 0 | - | 0 | 0 |
|  | Mucositis oral | | - | 0 | 0 | - | 5 (10.2) | 0 | - | 5 (62.5) | 0 | 4 (20) | 1 (5) | 0 |
|  | Constipation | | 7 (28) | 0 | 0 | 14 (28.6) | 0 | 0 | 3 (37.5) | 0 | 0 | - | 0 | 0 |
|  | Colonic perforation | | - | 1 (4) | 0 | - | 0 | 0 | - | 0 | 0 | - | 0 | 0 |
|  | Diarrhoea | | 9 (36) | 1 (4) | 0 | 12 (24.5) | 5 (10.2) | 0 | 3 (37.5) | 1 (12.5) | 0 | - | 1 (5) | 0 |
|  | Gastric haemorrhage | | - | 1 (4) | 0 | - | 0 | 0 | - | 0 | 0 | - | 0 | 0 |
|  | Other - tonsillar abscess | | - | 0 | 0 | - | 1 (2) | 0 | - | 0 | 0 | - | 0 | 0 |
| **General disorders and administration site conditions** | | | **15 (60)** | **4 (16)** | **0** | **24 (49)** | **9 (18.4)** | **0** | **3 (37.5)** | **3 (37.5)** | **0** | **6 (30)** | **1 (5)** | **0** |
|  | Fever | | 6 (24) | 1 (4) | 0 | 11 (22.4) | 3 (6.1) | 0 | 3 (37.5) | 1 (12.5) | 0 | - | 0 | 0 |
|  | Non-cardiac chest pain | | - | 1 (4) | 0 | - | 0 | 0 | - | 0 | 0 | - | 0 | 0 |
|  | Fatigue | | 10 (40) | 1 (4) | 0 | 20 (40.8) | 3 (6.1) | 0 | 3 (37.5) | 1 (12.5) | 0 | 5 (25) | 0 | 0 |
|  | Facial pain | | - | 0 | 0 | - | 1 (2) | 0 | - | 0 | 0 | - | 0 | 0 |
|  | Pain | | 5 (20) | 1 (4) | 0 | 10 (20.4) | 2 (4.1) | 0 | 2 (25.0) | 1 (12.5) | 0 | - | 1 (5) | 0 |
|  | Oedema limb | | 6 (24) | 0 | 0 | - | 0 | 0 | 2 (25.0) | 0 | 0 | - | 0 | 0 |
| **Hepatobiliary disorders** | | | **-** | **0** | **0** | **-** | **2 (4.1)** | **0** | **-** | **0** | **0** | **-** | **0** | **0** |
|  | GGT increased | | - | 0 | 0 | - | 1 (2) | 0 | - | 0 | 0 | - | 0 | 0 |
|  | Hepatic failure | | - | 0 | 0 | - | 1 (2) | 0 | - | 0 | 0 | - | 0 | 0 |
| **Infections and infestations** | | | **7 (28)** | **5 (20)** | **0** | **10 (20.4)** | **16 (32.7)** | **2 (4.1)** | **-** | **6 (75.0)** | **1 (12.5)** | **-** | **10 (50)** | **0** |
|  | Upper respiratory infection | | - | 0 | 0 | - | 1 (2) | 0 | - | 1 (12.5) | 0 | - | 0 | 0 |
|  | Sepsis | | - | 0 | 0 | - | 7 (14.3) | 0 | - | 4 (50) | 0 | - | 3 (15) | 0 |
|  | Pneumonia | | - | 0 | 0 | - | 3 (6.1) | 0 | - | 0 | 0 | - | 0 | 0 |
|  | Lung infection | | - | 0 | 0 | - | 2 (4.1) | 2 (4.1) | - | 0 | 1 (12.5) | - | 2 (10) | 0 |
|  | Urinary tract infection | | - | 1 (4) | 0 | - | 0 | 0 | - | 1 (12.5) | 0 | - | 1 (5) | 0 |
|  | Pharyngitis | | - | 0 | 0 | - | 0 | 0 | - | 1 (12.5) | 0 | - | 0 | 0 |
|  | Device related infection | | - | 1 (4) | 0 | - | 2 (4.1) | 0 | - | 2 (25) | 0 | - | 1 (5) | 0 |
|  | Skin infection | | - | 0 | 0 | - | 2 (4.1) | 0 | - | 0 | 0 | - | 2 (10) | 0 |
|  | Kidney infection | | - | 1 (4) | 0 | - | 0 | 0 | - | 0 | 0 | - | 0 | 0 |
|  | Other - chest infection | | - | 0 | 0 | - | 1 (2) | 0 | - | 0 | 0 | - | 0 | 0 |
|  | Other-bacterial - coag negative staph | | - | 0 | 0 | - | 1 (2) | 0 | - | 0 | 0 | - | 0 | 0 |
|  | Other-viral - NOS-inf | | - | 0 | 0 | - | 1 (2) | 0 | - | 0 | 0 | - | 1 (5) | 0 |
|  | Other-bacterial - enterococcus | | - | 0 | 0 | - | 1 (2) | 0 | - | 0 | 0 | - | 0 | 0 |
|  | Other-bacterial - NOS-inf | | - | 1 (4) | 0 | - | 3 (6.1) | 0 | - | 2 (25) | 0 | - | 1 (5) | 0 |
|  | Other - diverticulitis | | - | 1 (4) | 0 | - | 0 | 0 | - | 0 | 0 | - | 0 | 0 |
|  | Other-fungal - NOS-inf | | - | 1 (4) | 0 | - | 1 (2) | 0 | - | 1 (12.5) | 0 | - | 0 | 0 |
|  | Other - oesophageal candida | | - | 0 | 0 | - | 0 | 0 | - | 1 (12.5) | 0 | - | 0 | 0 |
|  | Other-viral - parainfluenza | | - | 0 | 0 | - | 0 | 0 | - | 0 | 0 | - | 1 (5) | 0 |
|  | Other - abdominal infection | | - | 0 | 0 | - | 0 | 0 | - | 0 | 0 | - | 1 (5) | 0 |
|  | Other - unknown origin | | - | 0 | 0 | - | 1 (2) | 0 | - | 0 | 0 | - | 0 | 0 |
| **Injury, poisoning and procedural complications** | | | **-** | **2 (8)** | **0** | **-** | **1 (2)** | **0** | **-** | **0** | **0** | **-** | **0** | **0** |
|  | Spinal fracture | | - | 0 | 0 | - | 1 (2) | 0 | - | 0 | 0 | - | 0 | 0 |
|  | Vascular access complication | | - | 1 (4) | 0 | - | 0 | 0 | - | 0 | 0 | - | 0 | 0 |
|  | Injury poisoning and procedural complications - other(bilateral hypodense subdural collections) | | - | 1 (4) | 0 | - | 0 | 0 | - | 0 | 0 | - | 0 | 0 |
| **Investigations** | | | **-** | **21 (84)** | **0** | **-** | **45 (91.8)** | **0** | **-** | **7 (87.5)** | **0** | **-** | **15 (75)** | **0** |
|  | ALT increased | | 10 (40) | 1 (4) | 0 | 16 (32.7) | 9 (18.4) | 0 | 2 (25) | 1 (12.5) | 0 | - | 1 (5) | 0 |
|  | Platelet count decreased | | 6 (24) | 11 (44) | 0 | - | 36 (73.5) | 0 | - | 5 (62.5) | 0 | - | 12 (60) | 0 |
|  | AST increased | | - | 0 | 0 | - | 3 (6.1) | 0 | 2 (25) | 0 | 0 | - | 0 | 0 |
|  | Alk Phos increased | | 9 (36) | 0 | 0 | 15 (30.6) | 1 (2) | 0 | - | 1 (12.5) | 0 | - | 0 | 0 |
|  | Urine output decreased | | - | 0 | 0 | - | 1 (2) | 0 | - | 1 (12.5) | 0 | - | 0 | 0 |
|  | White blood cell decreased | | - | 19 (76) | 0 | - | 42 (85.7) | 0 | - | 6 (75) | 0 | - | 12 (60) | 0 |
|  | Weight loss | | 5 (20) | 1 (4) | 0 | - | 1 (2) | 0 | 2 (25) | 0 | 0 | - | 0 | 0 |
|  | Creatinine increase | | 6 (24) | 2 (8) | 0 | - | 1 (2) | 0 | 2 (25) | 0 | 0 | - | 0 | 0 |
|  | Electrocardiogram qt corrected interval prolonged | | - | 1 (4) | 0 | - | 0 | 0 | - | 0 | 0 | - | 0 | 0 |
|  | Neutrophil decreased | | - | 21 (84) | 0 | - | 38 (77.6) | 0 | - | 6 (75) | 0 | - | 15 (75) | 0 |
|  | Lymphocyte decreased | | - | 2 (8) | 0 | - | 2 (4.1) | 0 | - | 2 (25) | 0 | - | 0 | 0 |
|  | Blood bilirubin increased | | 6 (24) | 0 | 0 | - | 2 (4.1) | 0 | 2 (25) | 1 (12.5) | 0 | - | 1 (5) | 0 |
| **Metabolism and nutrition disorders** | | | 5 (20) | 5 (20) | 0 | 10 (20.4) | 5 (10.2) | 0 | 2 (25) | 2 (25) | 0 | - | 1 (5) | 0 |
|  | Hypoalbuminemia | | 6 (24) | 1 (4) | 0 | - | 0 | 0 | - | 1 (12.5) | 0 | - | 0 | 0 |
|  | Hypocalcaemia | | - | 0 | 0 | - | 0 | 0 | - | 1 (12.5) | 0 | - | 0 | 0 |
|  | Hypokalaemia | | - | 1 (4) | 0 | - | 2 (4.1) | 0 | - | 2 (25) | 0 | - | 0 | 0 |
|  | Alkalosis | | - | 0 | 0 | - | 0 | 0 | - | 1 (12.5) | 0 | - | 0 | 0 |
|  | Hyperglycaemia | | - | 1 (4) | 0 | - | 1 (2) | 0 | - | 0 | 0 | - | 1 (5) | 0 |
|  | Anorexia | | - | 0 | 0 | - | 1 (2) | 0 | 2 (25.0) | 0 | 0 | - | 0 | 0 |
|  | Hypophosphatemia | | - | 1 (4) | 0 | - | 0 | 0 | - | 1 (12.5) | 0 | - | 0 | 0 |
|  | Dehydration | | - | 2 (8) | 0 | - | 1 (2) | 0 | - | 0 | 0 | - | 0 | 0 |
|  | Tumour lysis syndrome | | - | 0 | 0 | - | 2 (4.1) | 0 | - | 0 | 0 | - | 0 | 0 |
|  | Hyponatremia | | - | 1 (4) | 0 | - | 1 (2) | 0 | - | 0 | 0 | - | 0 | 0 |
| **Musculoskeletal and connective tissue disorders** | | | **5 (20)** | **0** | **0** | **-** | **1 (2)** | **0** | **-** | **0** | **0** | **-** | **0** | **0** |
|  | Back pain | | - | 0 | 0 | - | 1 (2) | 0 | - | 0 | 0 | - | 0 | 0 |
| **Nervous system disorders** | | | **9 (36)** | **3 (12)** | **0** | **15 (30.6)** | **6 (12.2)** | **0** | **3 (37.5)** | **2 (25)** | **0** | **-** | **1 (5)** | **0** |
|  | Intracranial haemorrhage | | - | 0 | 0 | - | 0 | 0 | - | 0 | 0 | - | 1 (5) | 0 |
|  | Peripheral sensory neuropathy | | - | 0 | 0 | 10 (20.4) | 2 (4.1) | 0 | - | 0 | 0 | - | 0 | 0 |
|  | Cerebrospinal fluid leakage | | - | 1 (4) | 0 | - | 0 | 0 | - | 0 | 0 | - | 0 | 0 |
|  | Stroke | | - | 0 | 0 | - | 1 (2) | 0 | - | 0 | 0 | - | 0 | 0 |
|  | Intracranial haemorrhage | | - | 0 | 0 | - | 0 | 0 | - | 0 | 0 | - | 1 (5) | 0 |
|  | Encephalopathy | | - | 0 | 0 | - | 1 (2) | 0 | - | 0 | 0 | - | 0 | 0 |
|  | Vasovagal reaction | | - | 0 | 0 | - | 0 | 0 | - | 1 (12.5) | 0 | - | 0 | 0 |
|  | Headache | | 5 (20) | 2 (8) | 0 | 10 (20.4) | 2 (4.1) | 0 | 2 (25) | 0 | 0 | - | 0 | 0 |
|  | Depressed level of consciousness | | - | 0 | 0 | - | 0 | 0 | - | 1 (12.5) | 0 | - | 0 | 0 |
|  | Other - bilateral hyperdense subdural collections | | - | 1 (4) | 0 | - | 0 | 0 | - | 0 | 0 | - | 0 | 0 |
| **Psychiatric disorders** | | | **-** | **1 (4)** | **0** | **-** | **1 (2)** | **0** | **-** | **1 (12.5)** | **0** | **-** | **0** | **0** |
|  | Confusion | | - | 0 | 0 | - | 1 (2) | 0 | - | 0 | 0 | - | 0 | 0 |
|  | Delirium | | - | 0 | 0 | - | 0 | 0 | - | 1 (12.5) | 0 | - | 0 | 0 |
|  | Depression | | - | 1 (4) | 0 | - | 0 | 0 | - | 0 | 0 | - | 0 | 0 |
| **Renal and urinary disorders** | | | **-** | **2 (8)** | **0** | **-** | **1 (2)** | **0** | **2 (25)** | **1 (12.5)** | **0** | **-** | **0** | **0** |
|  | Acute kidney injury | | - | 2 (8) | 0 | - | 1 (2) | 0 | - | 1 (12.5) | 0 | - | 0 | 0 |
| **Respiratory, thoracic and mediastinal disorders** | | | **-** | **1 (4)** | **0** | **11 (22.4)** | **2 (4.1)** | **0** | **2 (25)** | **2 (25)** | **0** | **-** | **0** | **0** |
|  | Cough | | - | 0 | 0 | - | 0 | 0 | - | 1 (12.5) | 0 | - | 0 | 0 |
|  | Hypoxia | | - | 1 (4) | 0 | - | 1 (2) | 0 | - | 1 (12.5) | 0 | - | 0 | 0 |
|  | Pulmonary oedema | | - | 1 (4) | 0 | - | 0 | 0 | - | 1 (12.5) | 0 | - | 0 | 0 |
|  | Respiratory failure | | - | 0 | 0 | - | 1 (2) | 0 | - | 0 | 0 | - | 0 | 0 |
|  | Sore throat | | - | 0 | 0 | - | 0 | 0 | - | 1 (12.5) | 0 | - | 0 | 0 |
|  | Dyspnoea | | - | 1 (4) | 0 | - | 0 | 0 | - | 1 (12.5) | 0 | - | 0 | 0 |
| **Skin and subcutaneous tissue disorders** | | | **-** | **0** | **0** | **-** | **1 (2)** | **0** | **-** | **0** | **0** | **-** | **0** | **0** |
|  | Rash maculo-papular | | - | 0 | 0 | - | 1 (2) | 0 | - | 0 | 0 | - | 0 | 0 |
| **Vascular disorders** | | | **-** | **2 (8)** | **0** | **-** | **3 (6.1)** | **0** | **-** | **0** | **0** | **-** | **2 (10)** | **0** |
|  | Thromboembolic event | | - | 1 (4) | 0 | - | 1 (2) | 0 | - | 0 | 0 | - | 1 (5) | 0 |
|  | Hypotension | | - | 0 | 0 | - | 1 (2) | 0 | - | 0 | 0 | - | 0 | 0 |
|  | Hypertension | | - | 0 | 0 | - | 1 (2) | 0 | - | 0 | 0 | - | 1 (5) | 0 |
|  | Other - extension of pre-existing DVT | | - | 1 (4) | 0 | - | 0 | 0 | - | 0 | 0 | - | 0 | 0 |
| **Any AE** | | | **-** | **22 (88)** | **0** | **-** | **45 (91.8)** | **3 (6.1)** | **-** | **7 (87.5)** | **1 (12.5)** | **-** | **19 (95)** | **1 (5)** |
|  | | |  |  |  |  |  |  |  |  |  |  |  |  |
| **Overview by treatment block** | | |  |  |  |  |  |  |  |  |  |  |  |  |
|  | | |  | | |  | | |  | | |  | | |
| **Induction 1*** | | | N=25 | | | N=49 | | | N=8 | | | N=20 | | |
|  | | Any grade 3-4 AE, N (%) | 22 (88) | | | 48 (98) | | | 8 (100) | | | 20 (10) | | |
|  | | Median number of grade 3-4 AEs (IQR) | 9(6- 11) | | | 10(8- 13) | | | 16(8- 20.5) | | | 6.5(5.5- 10) | | |
| **Induction 2*** | | | N=22 | | | N=34 | | | N=4 | | | N=16 | | |
|  | | Any grade 3-4 AE, N (%) | 19 (86.4) | | | 33 (97.1) | | | 4 (100) | | | 16 (100) | | |
|  | | Median number of grade 3-4 AEs (IQR) | 6(3- 8) | | | 6(4- 10) | | | 15(9- 17.5) | | | 5.5(3.5-8.5) | | |
| **Consolidation 1*** | | | **N=19** | | | **N=30** | | | **N=4** | | | **N=11** | | |
|  | | Any grade 3-4 AE, N (%) | 17 (89.5) | | | 29 (96.7) | | | 4 (100) | | | 11 (100) | | |
|  | | Median number of grade 3-4 AEs (IQR) | 6(3- 7) | | | 6(4- 8) | | | 10.5(6- 14) | | | 3(3.0-7) | | |
|  | | |  | | |  | | |  | | |  | | |

*All grade 3-4 and grade 5 events are reported, grade 1-2 events that occurred in at least 20% of patients are reported. *Significant differences seen in numbers of AEs per patient: A vs C (p=0.045), B vs D (p=0.023) and C vs D (p=0.026) in induction 1, A vs C (p=0.030), B vs C (p=0.035) and C vs D (p=0.026) in induction 2 and C vs D (p=0.034) in consolidation 1.*

**Table S5 -EFS and OS for B and T ALL**

| Pathway | 1-year EFS | 2-years EFS | *3-year EFS | 1-years OS | 2-years OS | 3-years OS |
| --- | --- | --- | --- | --- | --- | --- |
| B-cell | 51.0% (40.9 – 60.2) | 30.4% (21.8 – 339.4) | 21.0% (13.6 – 29.5) | 60.8% (50.6 – 69.5) | 36.3% (27.1 – 45.5) | 25.8% (17.6 – 34.7) |
| **T-cell | 50.0% (22.9 – 72.2) | 14.3% (2.3 – 36.6) | - | 64.3% (34.3 – 83.3) | 21.4% (5.2 – 444.8) | - |

*No patient with T-ALL have been followed -up for 3 years so the 3 year rates are not available

** Only N=14 T-cell (1 pathway A, 11 B, 1 C and 1 reg only) , all but one have had an event - 9 relapses (all later died) and 4 deaths without relapse.

**Table S6: Comparison of baseline QOL by treatment pathway**

| **Baseline characteristic** | | **Pathway B** | **Pathway C** | **Pathway D** | **p-value**  **B vs C** | **p-value**  **B vs D** | **p-value**  **C vs D** |
| --- | --- | --- | --- | --- | --- | --- | --- |
|  |  | **N=51** | **N=9** | **N=21** |  |  |  |
|  |  | **N (%)** | **N (%)** | **N (%)** |  |  |  |
|  |  |  |  |  |  |  |  |
| **QLQ-C30** | | **N=43** | **N=7** | **N=19** |  |  |  |
|  | Global heath QOL score, median (IQR | 41.7(16.7 - 66.7) | 66.7(25 - 83.3) | 50(33.3 - 50) | 0.34 | 0.83 | 0.48 |
| **Functional Scales** | |  |  |  |  |  |  |
|  | Physical functioning | 86.7(66.7 - 100) | 80(26.7 - 100) | 60(53.3 - 80) | 0.62 | 0.014 | 0.49 |
|  | Role functioning | 66.7(33.3 - 100) | 33.3(16.7 - 100) | 66.7(16.7 - 83.3) | 0.35 | 0.40 | 0.73 |
|  | Emotional functioning | 75(58.3 - 83.3) | 58.3(33.3 - 75) | 75(50 - 91.7) | 0.14 | 0.98 | 0.18 |
|  | Cognitive functioning | 100(83.3 - 100) | 83.3(66.7 - 100) | 83.3(66.7 - 100) | 0.42 | 0.16 | 0.90 |
|  | Social functioning | 66.7(33.3 - 100) | 100.0(16.7 - 100) | 50(16.7 - 66.7) |  |  |  |
| **Symptom scales** | |  |  |  |  |  |  |
|  | Fatigue | 33.3(22.2 - 66.7) | 66.7(33.3 - 88.9) | 55.6(33.3 - 77.8) | 0.17 | 0.12 | 0.64 |
|  | Nausea and Vomiting | 0(0 - 16.7) | 0.0(0 - 16.7) | 0(0 - 33.3) | 0.69 | 0.66 | 0.62 |
|  | Pain | 0(0 - 33.3) | 16.7(0 - 33.3) | 0(0 - 33.3) | 0.99 | 0.93 | 0.85 |
|  | Dyspnoea | 33.3(0 - 66.7) | 33.3(0 - 100) | 66.7(33.3 - 100) | 0.83 | 0.023 | 0.39 |
|  | Insomnia | 33.3(0 - 33.3) | 33.3(0 - 66.7) | 33.3(0 - 66.7) | 0.96 | 0.14 | 0.37 |
|  | Appetite loss | 0(0 - 33.3) | 33.3(0 - 66.7) | 33.3(0 - 100) | 0.26 | 0.042 | 0.65 |
|  | Constipation | 0(0 - 33.3) | 0(0 - 33.3) | 0(0 - 0) | 0.66 | 0.38 | 0.81 |
|  | Diarrhoea | 0(0 - 33.3) | 0(0 - 0) | 0(0 - 33.3) | 0.29 | 0.85 | 0.26 |
|  | Financial | 0(0 - 0) | 0(0 - 0) | 0(0 - 33.3) | 0.89 | 0.31 | 0.48 |
| **FACTIT** | |  |  |  |  |  |  |
|  | Physical well-being | 23(17 - 25.7) | 22(21 - 27) | 22(18 - 24) | 0.65 | 0.50 | 0.60 |
|  | Social/family well-being | 25(22.4 - 28) | 23.3(15 - 28) | 24.5(22 - 27) | 0.66 | 0.77 | 0.73 |
|  | Emotional well-being | 15.5(12 - 19) | 17.0(9 - 22) | 18.5(17 - 20) | 0.92 | 0.037 | 0.63 |
|  | Functional well-being | 14(10.5 - 21.5) | 14.0(10 - 21) | 14.5(12.8 - 22.4) | 0.83 | 0.80 | 0.76 |
|  | FACT-G total score | 76.5(67.8 - 85.9) | 65.0(58.8 - 89) | 80.7(70 - 85) | 0.64 | 0.71 | 0.35 |
|  |  |  |  |  |  |  |  |
|  | Leukaemia subscale | 46(41 - 54.2) | 47(38 - 49) | 43(41 - 55) | 0.50 | 0.61 | 0.70 |
|  | Leukaemia trial outcome index | 83.3(69.2 - 100.5) | 82(66 - 95) | 84(75.5 - 94.8) | 0.71 | 0.97 | 0.80 |
|  | Leukaemia total | 121.1(106.8 - 139.1) | 112(96.8 - 136) | 125(114.5 - 138.3) | 0.60 | 0.61 | 0.43 |
|  |  |  |  |  |  |  |  |
|  | Fatigue subscale | 38(25 - 45.8) | 36(17 - 42) | 33.5(19 - 40.1) | 0.40 | 0.20 | 0.90 |
|  | Fatigue trial outcome index | 77(56 - 89) | 66(51 - 90) | 74(50 - 83) | 0.69 | 0.51 | 0.95 |
|  | Fatigue total | 114(90 - 127.3) | 107(75.8 - 114) | 119(89 - 127) | 0.38 | 0.94 | 0.22 |

**Table S7: Baseline comorbidities by pathway**

| **Comorbidities** | | **Pathway A** | **Pathway B** | **Pathway C** | **Pathway D** | **Pathway E** | **All** | **UKALL 14 cohort** |
| --- | --- | --- | --- | --- | --- | --- | --- | --- |
|  |  | **N=25** | **N=51** | **N=9** | **N=21** | **N=15** | **N=121** | **N=65** |
|  |  | **N (%)** | **N (%)** | **N (%)** | **N (%)** | **N (%)** | **N (%)** | **N(%)** |
|  |  |  |  |  |  |  |  |  |
| **Charleson categories** | |  |  |  |  |  |  |  |
|  | Leukaemia | 25 (100) | 51 (100) | 9 (100) | 20 (95.2) | 15 (100) | 120 (99.2) | - |
|  | Diabetes | 3 (10) | 5 (9.8) | 1 (11.1) | 6 (28.6) | 2 (13.3) | 17 (14) | - |
|  | Mild Liver Disease | 2 (8) | 6 (11.8) | 1 (11.1) | 1 (4.8) | 0 | 10 (8.3) | - |
|  | Chronic Pulmonary Disease | 0 | 3 (5.9) | 1 (11.1) | 2 (9.5) | 2 (13.3) | 8 (6.6) | - |
|  | Moderate or severe renal disease | 2 (8) | 3 (5.9) | 0 | 1 (4.8) | 2 (13.3) | 8 (6.6) | - |
|  | Myocardial infarction | 4 (10) | 2 (3.9) | 0 | 0 | 1 (6.7) | 7 (5.8) | - |
|  | Congestive Heart Failure | 3 (12) | 1 (2) | 0 | 1 (4.8) | 1 (6.7) | 6 (5) | - |
|  | Cerebrovascular Disease | 1 (4) | 1 (2) | 0 | 1 (4.8) | 3 (20.0) | 6 (5) | - |
|  | Tumour without metastasis | 2 (8) | 2 (3.9) | 0 | 0 | 1 (6.7) | 5 (4.1) | - |
|  | Diabetes with end organ damage | 1 (4) | 0 | 0 | 0 | 3 (20.0) | 4 (3.3) | - |
|  | Connective Tissue Disease | 1 (4) | 0 | 0 | 0 | 1 (6.7) | 2 (1.7) | - |
|  | Peripheral Vascular Disease | 1 (4) | 0 | 0 | 0 | 1 (6.7) | 2 (1.7) | - |
|  | Lymphoma | 1 (4) | 1 (2) | 0 | 0 | 0 | 2 (1.7) | - |
|  | Dementia | 0 | 1 (2) | 0 | 0 | 0 | 1 (0.8) | - |
|  | Cerebrovascular disease | 0 | 1 (0) | 0 | 0 | 0 | 1 (0.8) | - |
|  | Metastatic solid tumour | 0 | 0 | 0 | 0 | 1 (6.7) | 1 (0.8) | - |
|  |  |  |  |  |  |  |  |  |
| **Baseline CTCAE events** | |  |  |  |  |  |  |  |
| **Blood and lymphatic system disorders** | | **1 (4)** | **3 (5.9)** | **1 (11.1)** | **3 (14.3)** | **0** | **8 (6.6)** | **1 (1.5)** |
|  | Anaemia | 1 (4) | 2 (3.9) | 1 (11.1) | 2 (9.5) | 0 | 6 (5) | 1 (1.5) |
|  | Febrile neutropenia | 0 | 0 | 1 (11.1) | 1 (4.8) | 0 | 2 (1.7) | 0 |
|  | Leucocytosis | 1 (4) | 0 | 0 | 0 | 0 | 1 (0.8) | 0 |
|  | Other: hemochromatosis | 0 | 1 (2) | 0 | 1 (4.8) | 0 | 2 (1.7) | 0 |
| **Cardiac disorders** | | **11 (44)** | **9 (17.6)** | **0** | **5 (23.8)** | **2 (13.3)** | **27 (22.3)** | **4 (6.2)** |
|  | Acute coronary syndrome | 3 (12) | 0 | 0 | 0 | 0 | 3 (2.5) | 0 |
|  | Atrial fibrillation | 0 | 2 (3.9) | 0 | 2 (9.5) | 1 (6.7) | 5 (4.1) | 2 (3.1) |
|  | Constrictive pericarditis | 0 | 0 | 0 | 1 (4.8) | 0 | 1 (0.8) | 0 |
|  | Heart failure | 2 (8) | 0 | 0 | 1 (4.8) | 0 | 3 (2.5) | 0 |
|  | Left ventricular systolic dysfunction | 2 (8) | 0 | 0 | 0 | 0 | 2 (1.7) | 0 |
|  | Myocardial infarction | 0 | 3 (5.9) | 0 | 0 | 1 (6.7) | 4 (3.3) | 0 |
|  | Supraventricular tachycardia | 0 | 0 | 0 | 0 | 0 | 0 | 1 (1.5) |
|  | Wolff-Parkinson-white syndrome | 1 (4) | 0 | 0 | 0 | 0 | 1 (0.8) | 0 |
|  | Other: right carotid endarterectomy | 0 | 1 (2.0) | 0 | 0 | 0 | 1 (0.8) | 0 |
|  | Other: carotid artery stenosis | 1 (4) | 0 | 0 | 0 | 0 | 1 (0.8) | 0 |
|  | Other: tachycardia NOS | 1 (4) | 0 | 0 | 0 | 0 | 1 (0.8) | 0 |
|  | Other : cardiovascular disease NOS | 1 (4) | 0 | 0 | 0 | 0 | 1 (0.8) | 0 |
|  | Other: coronary artery bypass graft | 1 (4) | 1 (2) | 0 | 1 (4.8) | 0 | 3 (2.5) | 0 |
|  | Other: pacemaker | 1 (4) | 0 | 0 | 0 | 0 | 1 (0.8) | 0 |
|  | Other: ischaemic heart disease | 2 (8) | 1 (2) | 0 | 1 (4.8) | 0 | 4 (3.3) | 0 |
|  | Other: angina | 0 | 3 (5.9) | 0 | 2 (9.5) | 0 | 5 (4.1) | 2 (3.1) |
| **Endocrine disorders** | | **3 (12)** | **4 (7.8)** | **0** | **2 (9.5)** | **1 (6.7)** | **10 (8.3)** | **4 (6.2)** |
|  | *Hyperthyroidism* | 0 | 0 | 0 | 0 | 0 | 0 | 1 (1.5) |
|  | Hypothyroidism | 3 (12) | 4 (7.8) | 0 | 2 (9.5) | 1 (6.7) | 10 (8.3) | 3 (4.6) |
| **Eye disorders** | | **1 (4)** | **0** | **0** | **3 (14.3)** | **2 (13.3)** | **6 (5)** | **3 (4.6)** |
|  | Cataract | 0 | 0 | 0 | 1 (4.8) | 1 (6.7) | 2 (1.7) | 2 (3.1) |
|  | Dry eye | 1 (4) | 0 | 0 | 0 | 0 | 1 (0.8) | 0 |
|  | Glaucoma | 1 (4) | 0 | 0 | 2 (9.5) | 0 | 3 (2.5) | 0 |
|  | Retinal vascular disorder | 0 | 0 | 0 | 0 | 0 | 0 | 0 |
|  | Other: ocular hypertension | 0 | 0 | 0 | 0 | 1 (6.7) | 1 (0.8) | 1 (1.5) |
| **Gastrointestinal disorders** | | **9 (36)** | **7 (13.7)** | **1 (11.1)** | **3 (14.3)** | **2 (13.3)** | **22 (18.2)** | **7 (10.8)** |
|  | Constipation | 2 (8) | 0 | 0 | 0 | 0 | 2 (1.7) | 0 |
|  | Diarrhoea | 3 (12) | 1 (2.) | 0 | 1 (4.8) | 0 | 5 (4.1) | 0 |
|  | Duodenal ulcer | 0 | 0 | 0 | 0 | 0 | 0 | 1 (1.5) |
|  | Dyspepsia | 0 | 1 (2.0) | 0 | 0 | 0 | 1 (0.8) | 0 |
|  | Gastritis | 1 (4) | 0 | 0 | 0 | 1 (6.7) | 2 (1.7) | 0 |
|  | Gastroesophageal reflux disease | 0 | 0 | 0 | 0 | 1 (6.7) | 1 (0.8) | 2 (3.1) |
|  | Haemorrhoids | 0 | 0 | 0 | 1 (4.8) | 0 | 1 (0.8) | 0 |
|  | Rectal haemorrhage | 0 | 0 | 0 | 1 (4.8) | 0 | 1 (0.8) | 0 |
|  | Other: reflux | 0 | 1 (2.0) | 0 | 0 | 0 | 1 (0.8) | 0 |
|  | Other: bowel ischemia | 0 | 0 | 0 | 1 (4.8) | 0 | 1 (0.8) | 0 |
|  | Other: Crohn's disease | 0 | 0 | 0 | 0 | 0 | 0 | 2 (3.1) |
|  | Other: diverticular disease/diverticulitis | 4 (16) | 2 (3.9) | 0 | 0 | 0 | 6 (5) | 1 (1.5) |
|  | Other: peptic ulcer disease | 0 | 0 | 0 | 0 | 0 | 0 | 0 |
|  | Other: hernia | 1 (4) | 2 (3.9) | 1 (11.1) | 1 (4.8) | 0 | 5 (4.1) | 1 (1.5) |
|  | Other: irritable bowel syndrome | 0 | 2 (3.9) | 0 | 0 | 0 | 2 (1.7) | 0 |
|  | Other: sigmoid volvulus | 0 | 0 | 0 | 0 | 0 | 0 | 1 (1.5) |
|  | Other: Ulcerative Colitis | 0 | 0 | 0 | 0 | 0 | 0 | 1 (1.5) |
| **General disorders and administration site conditions** | | **4 (16)** | **3 (5.9)** | **1 (11.1)** | **0** | **1 (6.7)** | **9 (7.4)** | **2 (3.1)** |
|  | Oedema limbs | 1 (4) | 0 | 0 | 0 | 0 | 1 (0.8) | 1 (1.5) |
|  | Fatigue | 3 (12) | 1 (2) | 0 | 0 | 0 | 4 (3.3) | 0 |
|  | Fever | 0 | 0 | 0 | 0 | 1 (6.7) | 1 (0.8) | 0 |
|  | Flu like symptoms | 1 (4) | 0 | 0 | 0 | 0 | 1 (0.8) | 0 |
|  | Infusion related reaction | 0 | 1 (2.0) | 0 | 0 | 0 | 1 (0.8) | 0 |
|  | Pain | 0 | 1 (2.0) | 1 (11.1) | 0 | 0 | 2 (1.7) | 0 |
|  | Other: sternum pain | 1 (4) | 0 | 0 | 0 | 0 | 1 (0.8) | 0 |
|  | Other: Fibromyalgia | 0 | 0 | 0 | 0 | 0 | 0 | 1 (1.5) |
| **Hepatobiliary disorders** | | **4 (16)** | **2 (3.9)** | **0** | **0** | **0** | **6 (5)** | 1 (1.5) |
|  | Cholecystitis | 0 | 1 (2.0) | 0 | 0 | 0 | 1 (0.8) | 0 |
|  | Other: cholecystitis | 0 | 1 (2.0) | 0 | 0 | 0 | 1 (0.8) | 0 |
|  | Other: gallstones | 1 (4) | 0 | 0 | 0 | 0 | 1 (0.8) | 0 |
|  | Other: cholangitis | 2 (8) | 0 | 0 | 0 | 0 | 2 (1.7) | 0 |
|  | Other: hepatobiliary disorders (NOS) | 1 (4) | 0 | 0 | 0 | 0 | 1 (0.8) | 0 |
|  | Other: Gilbert's syndrome | 0 | 0 | 0 | 0 | 0 | 0 | 1 (1.5) |
| **Immune system disorders** | | **2 (8)** | **0** | **0** | **0** | **0** | **2 (1.7)** | **3 (4.6)** |
|  | Other: penicillin allergy | 1 (4) | 0 | 0 | 0 | 0 | 1 (0.8) | 0 |
|  | Other: codeine allergy | 1 (4) | 0 | 0 | 0 | 0 | 1 (0.8) | 0 |
|  | Other: NSAIDS allergy | 1 (4) | 0 | 0 | 0 | 0 | 1 (0.8) | 0 |
|  | Other: cholestyramine allergy | 1 (4) | 0 | 0 | 0 | 0 | 1 (0.8) | 0 |
|  | Other: rosuvastatin allergy | 1 (4) | 0 | 0 | 0 | 0 | 1 (0.8) | 0 |
|  | Other: allergic to ampicillin | 1 (4) | 0 | 0 | 0 | 0 | 1 (0.8) | 0 |
|  | other: Autoimmune Neutropenia | 0 | 0 | 0 | 0 | 0 | 0 | 1 (1.5) |
|  | other: Common Variable Immunodeficiency | 0 | 0 | 0 | 0 | 0 | 0 | 1 (1.5) |
|  | other: hay fever | 0 | 0 | 0 | 0 | 0 | 0 | 1 (1.5) |
| **Infections and infestations** | | **3 (12)** | **4 (7.8)** | **3 (33.3)** | **2 (9.5)** | **1 (6.7)** | **13 (10.7)** | **3 (4.6)** |
|  | Abdominal infection | 0 | 1 (2) | 0 | 0 | 0 | 1 (0.8) | 0 |
|  | Gallbladder infection | 0 | 1 (2) | 0 | 1 (4.8) | 0 | 2 (1.7) | 0 |
|  | Hepatitis viral | 0 | 0 | 0 | 0 | 0 | 0 | 0 |
|  | Lung infection | 0 | 0 | 1 (11.1) | 1 (4.8) | 1 (6.7) | 3 (2.5) | 2 (3.1) |
|  | Phlebitis infective | 0 | 1 (2) | 0 | 0 | 0 | 1 (0.8) | 0 |
|  | Tooth infection | 0 | 0 | 1 (11.1) | 0 | 0 | 1 (0.8) | 0 |
|  | Urinary tract infection | 1 (4) | 0 | 0 | 0 | 0 | 1 (0.8) | 0 |
|  | Upper respiratory infection | 0 | 0 | 0 | 0 | 0 | 0 | 1 (1.5) |
|  | Other: shingles | 0 | 1 (2) | 0 | 0 | 0 | 1 (0.8) | 0 |
|  | Other: hepatitis c | 1 (4) | 0 | 0 | 0 | 0 | 1 (0.8) | 0 |
|  | Other: viral warts | 0 | 1 (2) | 0 | 0 | 0 | 1 (0.8) | 0 |
|  | Other: campylobacter infection | 0 | 0 | 1 (11.1) | 0 | 0 | 1 (0.8) | 0 |
|  | Other: syphilis | 1 (4) | 0 | 0 | 0 | 0 | 1 (0.8) | 0 |
| **Injury, poisoning and procedural complications** | | **0** | **1 (2)** | **0** | **0** | **0** | **1 (0.8)** | **1 (1.5)** |
|  | Fracture | 0 | 1 (2) | 0 | 0 | 0 | 1 (0.8) | 0 |
|  | other: Hydrogenic lung injury secondary to inhalation of epoxy resin | 0 | 0 | 0 | 0 | 0 | 0 | 1 (1.5) |
| **Investigations** | | **6 (24)** | **10 (19.6)** | **3 (33.3)** | **3 (14.3)** | **1 (6.7)** | **23 (19)** | **5 (7.7)** |
|  | Alkaline phosphatase increased | 1 (4) | 0 | 0 | 0 | 0 | 1 (0.8) | 1 (1.5) |
|  | Cholesterol high | 4 (16) | 6 (11.8) | 2 (22.2) | 2 (9.5) | 1 (6.7) | 15 (12.4) | 2 (3.1) |
|  | Creatinine increased | 1 (4) | 0 | 0 | 0 | 0 | 1 (0.8) | 1 (1.5) |
|  | Electrocardiogram qt corrected interval prolonged | 0 | 1 (2.0) | 0 | 0 | 0 | 1 (0.8) | 0 |
|  | GGT increased | 0 | 0 | 0 | 0 | 0 | 0 | 1 (1.5) |
|  | Lymphocyte count increased | 1 (4) | 1 (2.0) | 0 | 0 | 0 | 2 (1.7) | 1 (1.5) |
|  | Neutrophil count decreased | 0 | 1 (2.0) | 1 (11.1) | 1 (4.8) | 0 | 3 (2.5) | 2 (3.1) |
|  | Platelet count decreased | 0 | 3 (5.9) | 1 (11.1) | 1 (4.8) | 0 | 5 (4.1) | 1 (1.5) |
|  | Serum amylase increased | 1 (4) | 0 | 0 | 0 | 0 | 1 (0.8) | 0 |
|  | Weight loss | 2 (8) | 0 | 1 (11.1) | 0 | 0 | 3 (2.5) | 0 |
|  | White blood cell decreased | 0 | 1 (2) | 1 (11.1) | 1 (4.8) | 0 | 3 (2.5) | 0 |
| **Metabolism and nutrition disorders** | | **6 (24)** | **6 (11.8)** | **2 (22.2)** | **6 (28.6)** | **3 (20)** | **23 (19)** | **5 (7.7)** |
|  | Hyperglycaemia | 1 (4) | 0 | 0 | 0 | 2 (13.3) | 3 (2.5) | 0 |
|  | Hyperuricemia | 1 (4) | 0 | 0 | 0 | 0 | 1 (0.8) | 0 |
|  | Hypoalbuminemia | 1 (4) | 0 | 0 | 0 | 0 | 1 (0.8) | 0 |
|  | Hypokalaemia | 0 | 1 (2.0) | 0 | 0 | 0 | 1 (0.8) | 0 |
|  | Obesity | 0 | 0 | 1 (11.1) | 0 | 0 | 1 (0.8) | 0 |
|  | Other: diabetes | 3 (12) | 5 (9.8) | 2 (22.2) | 6 (28.6) | 1 (6.7) | 17 (14.0) | 5 (7.7) |
|  | Other: iron deficiency | 1 (4) | 0 | 0 | 0 | 0 | 1 (0.8) | 0 |
| **Musculoskeletal and connective tissue disorders** | | **9 (36)** | **9 (17.6)** | **4 (44.4)** | **3 (14.3)** | **2 (13.3)** | **27 (22.3)** | **10 (15.4)** |
|  | Arthritis | 2 (8.0) | 3 (5.9) | 3 (33.3) | 2 (9.5) | 0 | 10 (8.3) | 7 (10.8) |
|  | Avascular necrosis | 0 | 1 (2) | 0 | 0 | 0 | 1 (0.8) | 0 |
|  | Back pain | 1 (4) | 3 (5.9) | 1 (11.1) | 0 | 0 | 5 (4.1) | 1 (1.5) |
|  | Bone pain | 1 (4) | 0 | 0 | 0 | 0 | 1 (0.8) | 0 |
|  | Myalgia | 1 (4) | 0 | 0 | 0 | 0 | 1 (0.8) | 0 |
|  | Osteoporosis | 0 | 1 (2) | 0 | 0 | 2 (13.3) | 3 (2.5) | 1 (1.5) |
|  | Pain in extremity | 1 (4) | 0 | 0 | 0 | 0 | 1 (0.8) | 0 |
|  | Other: shoulder pain | 1 (4) | 0 | 0 | 0 | 0 | 1 (0.8) | 0 |
|  | Other: ankle pain | 1 (4) | 0 | 0 | 0 | 0 | 1 (0.8) | 0 |
|  | Other: gout | 1 (4) | 1 (2) | 0 | 0 | 0 | 2 (1.7) | 1 (1.5) |
|  | Other: polymyalgia rheumatica | 1 (4) | 0 | 0 | 0 | 0 | 1 (0.8) | 1 (1.5) |
|  | Other: Polyarthralgia | 0 | 0 | 0 | 0 | 0 | 0 | 1 (1.5) |
|  | Other: spondylosis | 1 (4) | 0 | 0 | 1 (4.8) | 0 | 2 (1.7) | 0 |
|  | Other: knee pain | 0 | 1 (2) | 0 | 0 | 0 | 1 (0.8) | 0 |
| **Neoplasms benign, malignant and unspecified (incl cysts and polyps)** | | **7 (28.0)** | **10 (19.6)** | **0** | **6 (28.6)** | **4 (26.7)** | **27 (22.3)** | **10 (15.4)** |
|  | **Malignant neoplasm** | **7 (28.0)** | **8 (15.7)** | **0** | **3 (14.3)** | **4 (26.7)** | **22 (18.1)** | **7 (10.8)** |
|  | Colorectal cancer | 1 (4) | 0 | 0 | 0 | 0 | 1 (0.8) | 1 (1.5) |
|  | Prostate cancer | 0 | 1 (2) | 0 | 1 (4.8) | 1 (6.7) | 3 (2.5) | 0 |
|  | Follicular lymphoma | 1 (4) | 0 | 0 | 0 | 0 | 1 (0.8) | 0 |
|  | Hepatocellular carcinoma | 0 | 1 (2) | 0 | 0 | 0 | 1 (0.8) | 0 |
|  | Myelodysplasia | 0 | 0 | 0 | 0 | 0 | 0 | 0 |
|  | ALL* | 0 | 1 (2) | 0 | 1 (4.8) | 0 | 2 (1.7) | 0 |
|  | AML | 0 | 0 | 0 | 1 (4.8) | 0 | 1 (0.8) | 0 |
|  | Myeloma | 0 | 1 (2) | 0 | 0 | 0 | 1 (0.8) | 0 |
|  | BCC | 0 | 1 (2) | 0 | 0 | 0 | 1 (0.8) | 0 |
|  | Breast cancer | 4 (16) | 1 (2) | 0 | 1 (4.8) | 2 (13.3) | 8 (6.6) | 3 (4.6) |
|  | CLL | 1 (4) | 0 | 0 | 0 | 0 | 1 (0.8) | 0 |
|  | Bladder cancer | 0 | 1 (2) | 0 | 0 | 0 | 1 (0.8) | 0 |
|  | Chronic myelomonocytic leukaemia | 0 | 0 | 0 | 0 | 1 (6.7) | 1 (0.8) | 0 |
|  | Mesothelioma | 0 | 1 (2) | 0 | 0 | 0 | 1 (0.8) | 0 |
|  | Melanoma (metastatic) | 0 | 0 | 0 | 0 | 0 | 0 | 1 (1.5) |
|  | Smouldering Myeloma | 0 | 0 | 0 | 0 | 0 | 0 | 1 (1.5) |
|  | Endometrioid adenomas & carcinomas | 0 | 0 | 0 | 0 | 0 | 0 | 1 (1.5) |
|  | **Benign neoplasm** | **0** | **2 (3.9)** | **0** | **3 (14.3)** | **0** | **5 (4.1)** | **3 (4.6)** |
|  | Other: benign prostatic hyperplasia | 0 | 2 (3.9) | 0 | 2 (9.5) | 0 | 4 (3.3) | 2 (3.1) |
|  | Other: benign ovarian cyst | 0 | 0 | 0 | 1 (4.8) | 0 | 1 (0.8) | 0 |
|  | Other: uterine fibroids | 0 | 0 | 0 | 1 (4.8) | 0 | 1 (0.8) | 0 |
|  | Other: Colonic polyp, benign | 0 | 0 | 0 | 0 | 0 | 0 | 1 (1.5) |
|  | **Not specified** | **0** | **1 (2.0)** | **0** | **0** | **0** | **1 (0.8)** | **0** |
|  | Other: mediastinal mass (most likely benign thymoma, but unconfirmed) | 0 | 1 (2.0) | 0 | 0 | 0 | 1 (0.8) | 0 |
| **Nervous system disorders** | | **5 (20)** | **4 (7.8)** | **0** | **2 (9.5)** | **3 (20)** | **14 (11.6)** | **5 (7.7)** |
|  | Lethargy | 1 (4) | 0 | 0 | 0 | 0 | 1 (0.8) | 0 |
|  | Stroke | 1 (4) | 1 (2) | 0 | 0 | 2 (13.3) | 4 (3.3) | 0 |
|  | Transient ischemic attacks | 0 | 1 (2) | 0 | 1 (4.8) | 0 | 2 (1.7) | 2 (3.1) |
|  | Tremor | 0 | 0 | 0 | 0 | 1 (6.7) | 1 (0.8) | 0 |
|  | Other: multiple sclerosis | 2 (8) | 0 | 0 | 0 | 0 | 2 (1.7) | 0 |
|  | Other: peripheral neuropathy NOS | 0 | 0 | 0 | 1 (4.8) | 0 | 1 (0.8) | 0 |
|  | Other: restless leg syndrome | 0 | 1 (2) | 0 | 0 | 0 | 1 (0.8) | 0 |
|  | Other: trigeminus neuralgia | 1 (4) | 0 | 0 | 0 | 0 | 1 (0.8) | 0 |
|  | Other: Guillen barre syndrome | 0 | 1 (2) | 0 | 0 | 0 | 1 (0.8) | 0 |
|  | Other: epilepsy | 0 | 0 | 0 | 0 | 0 | 0 | 2 (3.1) |
|  | Other: Sciatica | 0 | 0 | 0 | 0 | 0 | 0 | 1 (1.5) |
| **Non CTCAE** | | **0** | **2 (3.9)** | **0** | **0** | **0** | **2 (1.7)** | 0 |
|  | Other: cystitis NOS | 0 | 1 (2) | 0 | 0 | 0 | 1 (0.8) | 0 |
|  | Other: stent | 0 | 1 (2) | 0 | 0 | 0 | 1 (0.8) | 0 |
| **Psychiatric disorders** | | **4 (16)** | **3 (5.9)** | **2 (22.2)** | **1 (4.8)** | **0** | **10 (8.3)** | **4 (6.2)** |
|  | Anxiety | 1 (4) | 0 | 0 | 0 | 0 | 1 (0.8) | 1 (1.5) |
|  | Confusion | 1 (4) | 0 | 0 | 0 | 0 | 1 (0.8) | 0 |
|  | Depression | 1 (4) | 3 (5.9) | 2 (22.2) | 1 (4.8) | 0 | 7 (5.8) | 1 (1.5) |
|  | Insomnia | 1 (4) | 0 | 0 | 0 | 0 | 1 (0.8) | 0 |
|  | Psychosis | 0 | 0 | 0 | 0 | 0 | 0 | 1 (1.5) |
|  | Other: Alcohol consumption | 0 | 0 | 0 | 0 | 0 | 0 | 1 (1.5) |
| **Renal and urinary disorders** | | **2 (8)** | **4 (7.8)** | **0** | **2 (9.5)** | **1 (6.7)** | **9 (7.4)** | **2 (3.1)** |
|  | Abdominal pain | 0 | 0 | 0 | 0 | 1 (6.7) | 1 (0.8) | 0 |
|  | Acute kidney injury | 0 | 1 (2) | 0 | 0 | 0 | 1 (0.8) | 0 |
|  | Chronic kidney disease | 1 (4) | 3 (5.9) | 0 | 2 (9.5) | 0 | 6 (5.0) | 0 |
|  | Colitis | 1 (4) | 0 | 0 | 0 | 0 | 1 (0.8) | 0 |
|  | Urinary frequency | 1 (4) | 1 (2) | 0 | 0 | 0 | 2 (1.7) | 0 |
|  | Renal calculi | 0 | 0 | 0 | 0 | 0 | 0 | 1 (1.5) |
|  | Urinary retention | 0 | 0 | 0 | 0 | 0 | 0 | 1 (1.5) |
| **Reproductive system and breast disorders** | | **0** | **1 (2)** | **1 (11.1)** | **0** | **0** | **2 (1.7)** | **0** |
|  | Menorrhagia | 0 | 0 | 1 (11.1) | 0 | 0 | 1 (0.8) | 0 |
|  | Prostatic obstruction | 0 | 1 (2) | 0 | 0 | 0 | 1 (0.8) | 0 |
| **Respiratory, thoracic and mediastinal disorders** | | **3 (12)** | **5 (9.8)** | **1 (11.1)** | **4 (19)** | **3 (20)** | **16 (13.2)** | **6 (9.2)** |
|  | Dyspnoea | 1 (4) | 0 | 0 | 0 | 0 | 1 (0.8) | 0 |
|  | Sleep apnoea | 0 | 0 | 0 | 1 (4.8) | 2 (13.3) | 3 (2.5) | 0 |
|  | Pleural effusion |  |  |  |  |  |  | 1 (1.5) |
|  | Other: pulmonary fibrosis | 0 | 1 (2) | 0 | 0 | 0 | 1 (0.8) | 0 |
|  | Other: bronchiectasis | 1 (4) | 0 | 0 | 0 | 0 | 1 (0.8) | 0 |
|  | Other: asthma | 1 (4) | 1 (2) | 0 | 1 (4.8) | 1 (6.7) | 4 (3.3) | 2 (3.1) |
|  | Other: COPD | 0 | 3 (5.9) | 1 (11.1) | 2 (9.5) | 1 (6.7) | 7 (5.8) | 2 (3.1) |
|  | Other: Oesophageal Spasms | 0 | 0 | 0 | 0 | 0 | 0 | 1 (1.5) |
| **Skin and subcutaneous tissue disorders** | | **1 (4)** | **1 (2)** | **0** | **0** | **0** | **2 (1.7)** | **3 (4.6)** |
|  | Other: acne rosacea | 0 | 1 (2) | 0 | 0 | 0 | 1 (0.8) | 0 |
|  | Other: night sweats | 1 (4) | 0 | 0 | 0 | 0 | 1 (0.8) | 0 |
|  | other: Psoriasis | 0 | 0 | 0 | 0 | 0 | 0 | 3 (4.6) |
| **Surgical and medical procedures** | | **3 (12.0)** | **3 (5.9)** | **1 (11.1)** | **2 (9.5)** | **1 (6.7)** | **10 (8.3)** | **8 (12.3)** |
|  | Other: splenectomy, partial colectomy and pancreatectomy | 0 | 1 (2) | 0 | 0 | 0 | 1 (0.8) | 0 |
|  | Other: liver transplant | 2 (8.0) | 0 | 0 | 0 | 0 | 2 (1.7) | 0 |
|  | Other: knee replacement surgery | 0 | 0 | 0 | 0 | 0 | 0 | 0 |
|  | Other: vasectomy | 0 | 0 | 0 | 0 | 0 | 0 | 0 |
|  | Other: hysterectomy | 1 (4) | 1 (2.0) | 0 | 1 (4.8) | 1 (6.7) | 4 (3.3) | 4 (6.2) |
|  | Other: cholecystectomy | 1 (4) | 0 | 0 | 0 | 0 | 1 (0.8) | 2 (3.1) |
|  | Other: appendectomy | 1 (4) | 0 | 1 (11.1) | 0 | 1 (6.7) | 3 (2.5) | 1 (1.5) |
|  | Other: hernia | 1 (4) | 2 (3.9) | 1 (11.1) | 1 (4.8) | 0 | 5 (4.1) | 0 |
|  | Other: nephrectomy | 0 | 1 (2) | 0 | 0 | 0 | 1 (0.8) | 0 |
|  | Other: Bilateral hip replacement | 0 | 0 | 0 | 0 | 0 | 0 | 1 (1.5) |
|  | other: lumbar disc surgery | 0 | 0 | 0 | 0 | 0 | 0 | 1 (1.5) |
|  | other: tonsillectomy | 0 | 0 | 0 | 0 | 0 | 0 | 1 (1.5) |
| **Vascular disorders** | | **9 (36.0)** | **17 (33.3)** | **5 (55.6)** | **10 (47.6)** | **4 (26.7)** | **45 (37.2)** | **19 (29.2)** |
|  | Hematoma | 1 (4) | 0 | 0 | 0 | 0 | 1 (0.8) | 0 |
|  | Hypertension | 7 (28) | 16 (31.4) | 4 (44.4) | 9 (42.9) | 3 (20.0) | 39 (32.2) | 15 (23.1) |
|  | Hypotension | 1 (4.0) | 0 | 0 | 0 | 0 | 1 (0.8) | 0 |
|  | Lymphedema | 0 | 0 | 0 | 0 | 0 | 0 | 1 (1.5) |
|  | Superficial thrombophlebitis | 0 | 0 | 1 (11.1) | 1 (4.8) | 0 | 2 (1.7) | 0 |
|  | Thromboembolic event | 2 (8) | 1 (2) | 0 | 1 (4.8) | 1 (6.7) | 5 (4.1) | 4 (6.2) |
|  | Other: temporal arteritis | 0 | 0 | 0 | 1 (4.8) | 0 | 1 (0.8) | 0 |
| **Any** |  | 24 (96) | 41 (80.4) | 8 (88.9) | 18 (85.7) | 12 (80) | 103 (85.1) | 44 (67.7) |

*Both thought to be completely separate diagnoses rather than relapsed ALL (historical diagnoses at ages 34 and 65 with UKALL 60+ diagnoses at 56 and 81) TT

**Table S8: CIPN-20, FACT-LEU and FACT-FATIGUE scores by timepoint**

| **Measure** | **Induction 1** | **Induction 2** | **Consolidation 1** | **Maintenance 1** |
| --- | --- | --- | --- | --- |
|  | **Mean difference from baseline (99% CI)** | **Mean difference from baseline (99% CI)** | **Mean difference from baseline (99% CI)** | **Mean difference from baseline (99% CI)** |
|  |  |  |  |  |
| **CIPN** |  |  |  |  |
| **Sensory** | 4.74 (0.09 to 9.38)  p=0.009 | 8.88 (3.89 to 13.87)  p<0.001 | 10.13 (4.78 to 15.48)  p<0.001 | 10.81 (4.78 to 16.74)  p<0.001 |
| **Motor** | 3.87 (-0.91 to 8.65)  p=0.037 | 6.97 (1.82 to 12.12)  p<0.001 | 8.26 (2.75 to 13.78)  p<0.001 | 7.87 (1.76 to 13.99)  p=0.001 |
| **Autonomic** | 2.50 (-3.73 to 8.72)  p=0.30 | 0.75 (6.03 to 7.52)  p=0.78 | - 1. (-6.10 to 8.11)   p=0.72 | 0.17 (-7.92 to 8.25)  p=0.96 |
|  |  |  |  |  |
| **FACT** |  |  |  |  |
| **Physical wellbeing** | -0.47 (-2.12 to 1.19)  p=0.47 | -1.70 (-3.48 to 0.10)  p=0.015 | 0.93 (-0.96 to 2.82)  p=0.21 | 2.53 (0.39 to 4.67)  p=0.002 |
| **Social/family wellbeing** | 0.76 (-0.94 to 2.46)  p=0.25 | 0.52 (-1.32 to 2.36)  p=0.46 | 1.39 (-0.54 to 3.33)  p=0.064 | 2.06 (-0.13 to 4.25)  p=0.016 |
| **Emotional wellbeing** | 2.65 (1.21 to 4.10)  p<0.001 | 2.60 (1.04 to 4.16)  p<0.001 | 3.52 (1.87 to 5.17)  p<0.001 | 4.43 (2.59 to 6.26)  p<0.001 |
| **Functional wellbeing** | -2.89 (-5.00 to -0.59)  p=0.001 | -2.23 (-4.62 to 0.16)  p=0.16 | 1.19 (-1.33 -3.72)  p=0.22 | 2.92 (0.13 to 5.71)  p=0.007 |
| **FACT-G** | 0.27 (-0.41 to 0.47)  p=0.87 | -0.45 (-5.26 to 4.37)  p=0.81 | 7.40 (2.34 to 12.46)  p<0.001 | 12.62 (6.96 to 18.28)  p<0.001 |
| **FACT-Leu subscale** | 1.45 (-1.73 to 4.63)  p=0.24 | 0.21 (-3.21 to 3.62)  p=0.88 | 4.06 (0.41 – 7.71)  p=0.004 | 4.74 (0.66 to 8.83)  p=0.003 |
| **FACT-LEU TOI** | -1.88 (-7.46 to 3.70)  p=0.39 | -2.61 (-8.73 to 3.52)  p=0.27 | 6.29 (-0.13 to 12.71)  p=0.12 | 12.05 (4.83 to 19.28)  p<0.001 |
| **FACT-LEU total** | 1.61 (-5.06 to 8.28)  p=0.53 | 0.69 (-6.62 to 7.99)  p=0.81 | 11.39 (3.71 to 19.07)  p<0.001 | 17.73 (10.09 to 27.37)  p<0.001 |
| **FACT-F subscale** | -0.87 (-5.08 to 3.35)  p=60 | -2.31 (-6.88 to 2.25)  p=0.19 | 2.37 (-2.45 to 7.19)  p=0.21 | 5.84 (0.44 to 11.24)  p=0.005 |
| **FACT-F TOI** | -3.74 (-10.69 to 3.21)  p=0.17 | -4.51 (-12.15 to 3.12)  p=0.13 | 4.75 (-33.17 to 12.68)  p=0.12 | 12.80 (3.89 to 21.72)  P<0.001 |
| **FACT-F total** | -0.19 (-8.93 to 7.65)  p=0.95 | -0.90 (-9.51 to 7.71)  p=0.79 | 9.87 (0.92 to 18.82)  p=0.004 | 19.58 (9.52 – 29.64)  P<0.001 |
|  |  |  |  |  |

**Figure S1 Details of chemotherapy treatment, per pathway**

**
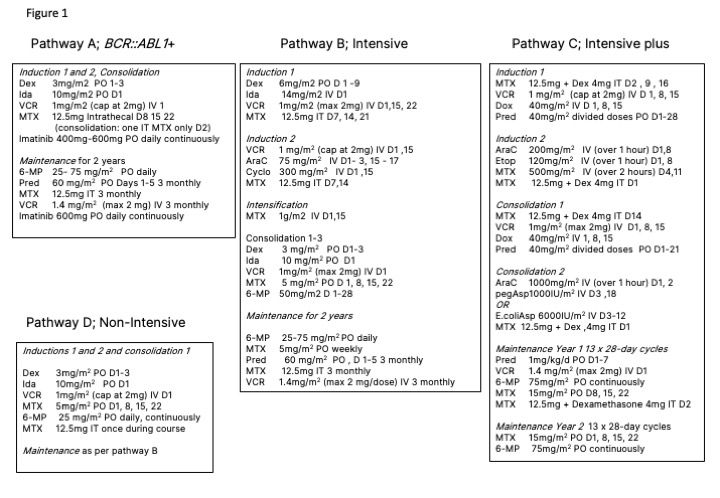
**

**Figure S2 Consort Diagram**

**
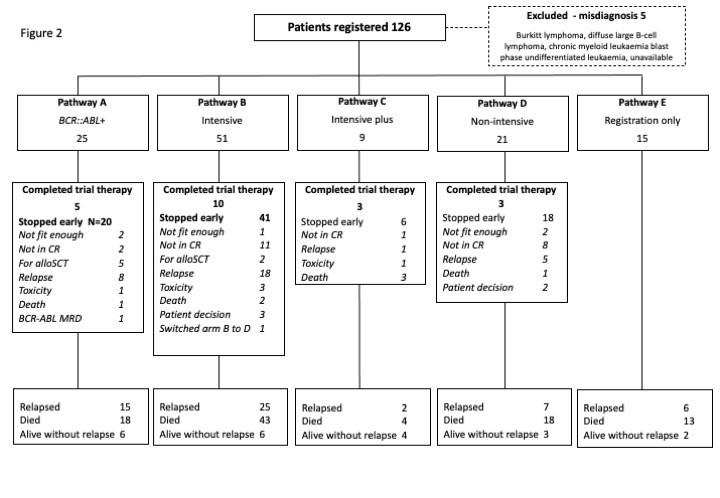
**

**Figure S3 A and B: EFS and OS by depth of response – CR and MRD**

**EFS**

**
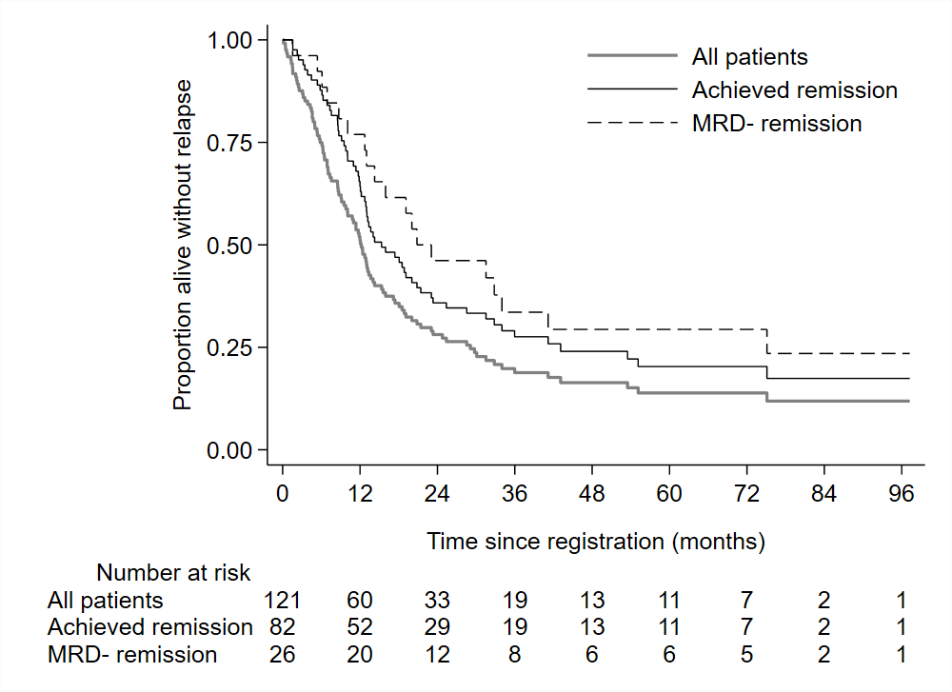
**

**OS**

**
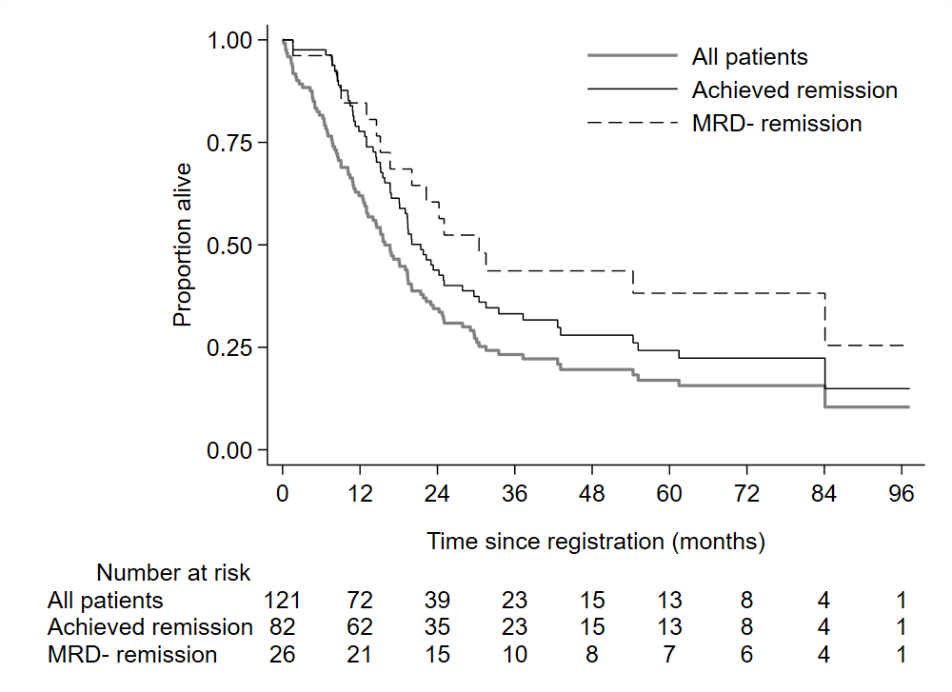
**

**Figures S4A -D**

S4A: QLQ-C30 Global heath score by pathway


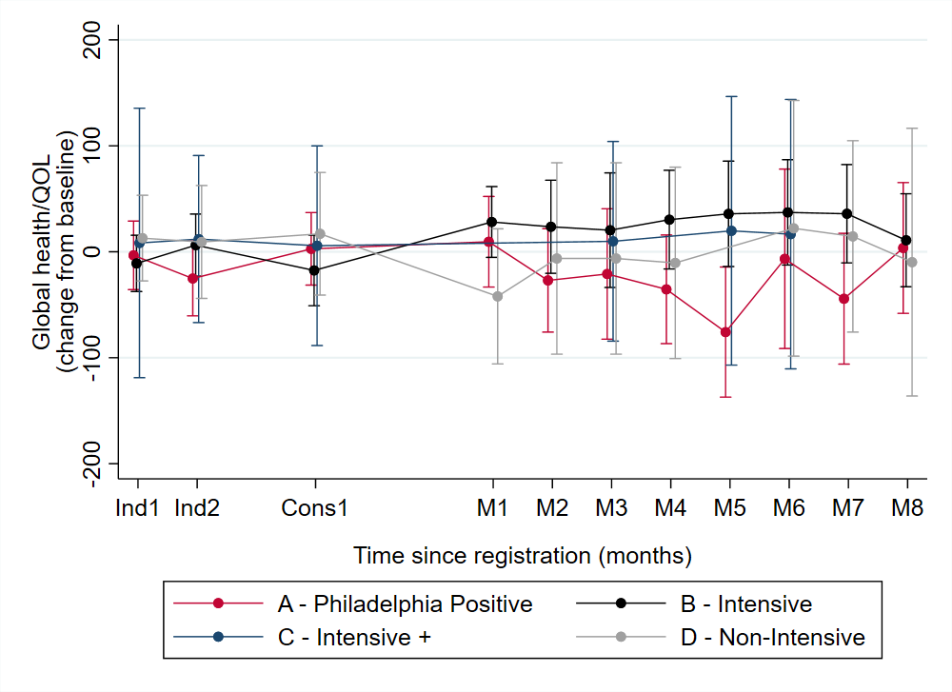


S4B: QLQ-C30- Physical functioning by pathway


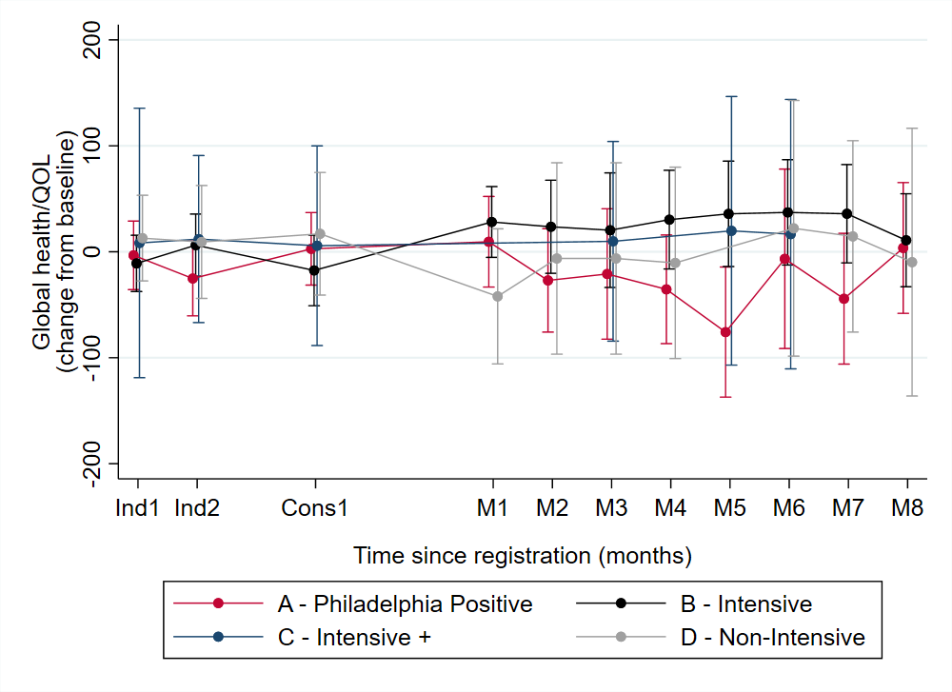


S4C: CIPN-20: Sensory scale


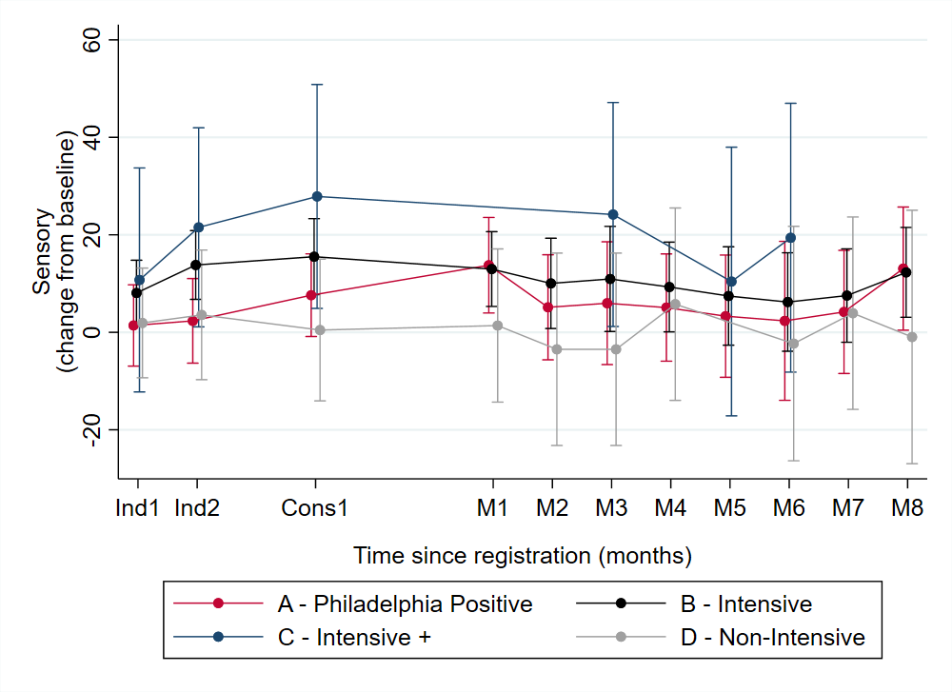


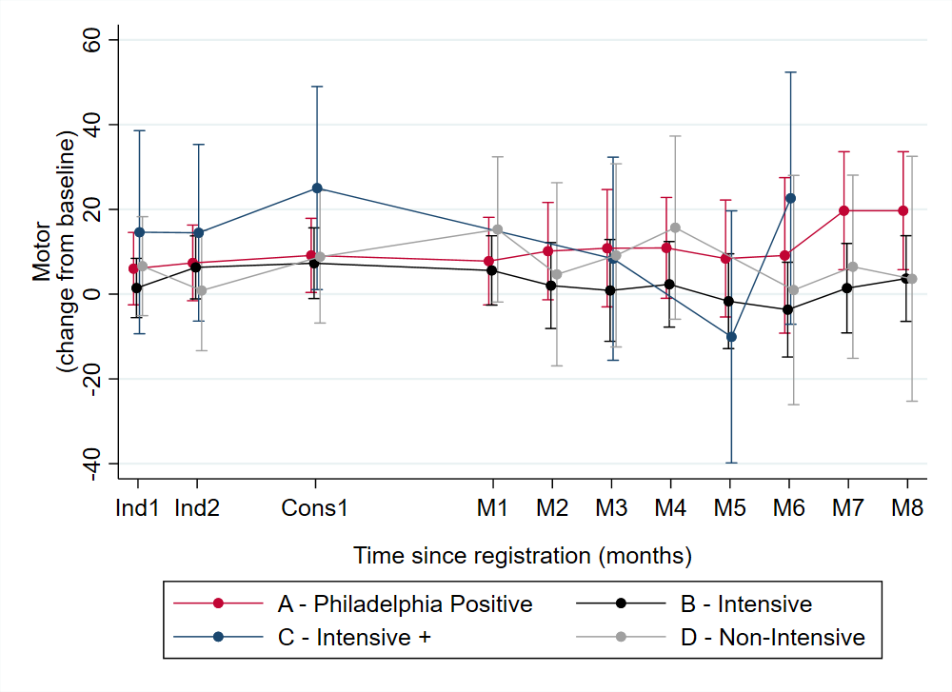


S4D: CIPN-20: Motor scale
